# Defining the Value of Future Research to Identify the Preferred Treatment of Meniscal Tear in the Presence of Knee Osteoarthritis

**DOI:** 10.1371/journal.pone.0130256

**Published:** 2015-06-18

**Authors:** Elena Losina, Elizabeth E. Dervan, A. David Paltiel, Yan Dong, R. John Wright, Kurt P. Spindler, Lisa A. Mandl, Morgan H. Jones, Robert G. Marx, Clare E. Safran-Norton, Jeffrey N. Katz

**Affiliations:** 1 Orthopaedic and Arthritis Center for Outcomes Research, Department of Orthopaedic Surgery, Brigham and Women’s Hospital, Boston, Massachusetts, United States of America; 2 Section of Clinical Sciences, Division of Rheumatology, Immunology and Allergy, Brigham and Women’s Hospital, Boston, Massachusetts, United States of America; 3 Harvard Medical School, Boston, Massachusetts, United States of America; 4 Department of Biostatistics, Boston University School of Public Health, Boston, Massachusetts, United States of America; 5 Yale School of Public Health and Yale School of Management, New Haven, Connecticut, United States of America; 6 Department of Orthopaedic Surgery, Brigham and Women’s Hospital, Boston, Massachusetts, United States of America; 7 Department of Orthopaedic Surgery, Cleveland Clinic, Garfield Heights, Ohio, United States of America; 8 Weill Cornell Medical College, New York, New York, United States of America; 9 Hospital for Special Surgery, New York, New York, United States of America; 10 Department of Rehabilitation Services, Brigham and Women’s Hospital, Boston, Massachusetts, United States of America; 11 Departments of Epidemiology and Environmental Health, Harvard T. H. Chan School of Public Health, Boston, Massachusetts, United States of America; Faculté de médecine de Nantes, FRANCE

## Abstract

**Background:**

Arthroscopic partial meniscectomy (APM) is extensively used to relieve pain in patients with symptomatic meniscal tear (MT) and knee osteoarthritis (OA). Recent studies have failed to show the superiority of APM compared to other treatments. We aim to examine whether existing evidence is sufficient to reject use of APM as a cost-effective treatment for MT+OA.

**Methods:**

We built a patient-level microsimulation using Monte Carlo methods and evaluated three strategies: Physical therapy (‘PT’) alone; PT followed by APM if subjects continued to experience pain (‘Delayed APM’); and ‘Immediate APM’. Our subject population was US adults with symptomatic MT and knee OA over a 10 year time horizon. We assessed treatment outcomes using societal costs, quality-adjusted life years (QALYs), and calculated incremental cost-effectiveness ratios (ICERs), incorporating productivity costs as a sensitivity analysis. We also conducted a value-of-information analysis using probabilistic sensitivity analyses.

**Results:**

Calculated ICERs were estimated to be $12,900/QALY for Delayed APM as compared to PT and $103,200/QALY for Immediate APM as compared to Delayed APM. In sensitivity analyses, inclusion of time costs made Delayed APM cost-saving as compared to PT. Improving efficacy of Delayed APM led to higher incremental costs and lower incremental effectiveness of Immediate APM in comparison to Delayed APM. Probabilistic sensitivity analyses indicated that PT had 3.0% probability of being cost-effective at a willingness-to-pay (WTP) threshold of $50,000/QALY. Delayed APM was cost effective 57.7% of the time at WTP = $50,000/QALY and 50.2% at WTP = $100,000/QALY. The probability of Immediate APM being cost-effective did not exceed 50% unless WTP exceeded $103,000/QALY.

**Conclusions:**

We conclude that current cost-effectiveness evidence does not support unqualified rejection of either Immediate or Delayed APM for the treatment of MT+OA. The amount to which society would be willing to pay for additional information on treatment outcomes greatly exceeds the cost of conducting another randomized controlled trial on APM.

## Introduction

Meniscal tear (MT) is a highly prevalent condition, particularly for individuals over age 50 and those with concomitant knee osteoarthritis (OA) [1]. Symptomatic MT is often treated surgically with arthroscopic partial meniscectomy (APM), a widespread procedure performed in over 350,000 people annually between the ages of 45 and 64 in the US [2].

Five recent randomized controlled trials (RCTs) have evaluated APM efficacy among symptomatic patients with MT and radiographic or pre-radiographic knee OA. One most recent study established the superiority of APM compared to non-operative management [3], while others failed to establish superiority of APM compared to non-operative management or sham procedures, demonstrating similar pain relief between surgical and non-surgical interventions [4–8]. In three of these studies, between 20% and 30% of patients originally assigned to the non-surgical arm crossed over to receive APM [3–6]. A recent meta-analysis concluded that over a short time horizon (6 months), APM is superior to non-operative management, but this superiority is not observed over a longer time horizon [9]. These data raise questions about the value of APM in patients with MT and knee OA. The high cross-over rates suggest that the most clinically effective treatment may be physical therapy (PT) followed by Delayed APM for those with persistent symptoms.

While these papers offer insight into the efficacy of APM vs. PT in persons with MT and OA, the economic implications of these treatments have not yet been evaluated. On the basis of these trials, some payers may consider revising reimbursement policies for APM. As various reimbursement policies are considered, a formal decision analysis may offer critical insight regarding the value of alternative treatment strategies.

We used decision analysis modeling informed by data from the Meniscal Tear in Osteoarthritis Research (MeTeOR) trial [6] to evaluate the long-term clinical and economic implications of alternative treatment strategies for symptomatic patients presenting with MT and osteoarthritic changes. Additionally, we used formal decision analysis and cost-effectiveness methods to determine the level of confidence regarding optimal decision choices and the value of further related research in this area.

## Methods

### Analytic Overview

We used a cost-effectiveness framework to evaluate three different treatment strategies: 1) physical therapy alone (‘PT’), 2) PT followed by APM only for subjects still symptomatic three months after physical therapy (‘Delayed APM’), and 3) APM offered as first line treatment to all subjects (‘Immediate APM’). Each strategy allowed subjects to proceed to total knee arthroplasty (TKA) if they demonstrated radiographic evidence of advanced knee OA as well as sustained pain.

We built a probabilistic Markov state-transition, computer-based simulation model in which a subject’s experience was described by a sequence of transitions among distinct health states. We evaluated the three strategies over ten years using quarterly cycles, meaning transitions between health states could occur in the model every three months of a subject’s life. Health states were defined by a subject’s knee pain status, treatment, and extent of radiographic progression. Health states were associated with distinct economic costs and decrements to health-related quality of life (QoL), outcomes which subjects accrued as they transitioned from one health state to another [10].

Subjects entered the model with knee OA at Kellgren-Lawrence (KL) grades 0,1, 2 or 3 [11]. A subject’s knee OA could progress structurally throughout the model, causing a subject to transition to a health state with potentially higher costs and lower QoL. Data for clinical and economic parameters were derived from the MeTeOR study for the first year after treatment and from national OA cohort studies for subsequent years.

To determine the value of different strategies for MT treatment, we conducted a cost-effectiveness analysis in accordance with recommendations of the US Panel of Cost-Effectiveness in Health and Medicine [12]. Preferences for health states were defined by ‘utilities’ that reflect societal preferences for different health states on a scale of 1.00 (perfect health) to 0.00 (death) [13]. Main outcomes for the analyses included quality-adjusted life years (QALYs) and costs accumulated over the 10-year lifespan. QALYs and costs were reported on a present-value basis using an annual discount rate of 3%. Cost-effectiveness for each treatment strategy was expressed in terms of incremental cost-effectiveness ratios (ICERs), or additional costs incurred for every QALY gained.

We employed probabilistic Monte Carlo simulation methods, which take into consideration uncertainty in the several key parameters. We used beta distributions to model the probabilities of failed pain relief, pain incidence and pain resolution post-treatment, and gamma distributions to model costs [14]. Further details on choice of parameter distributions are presented in the S1 Technical Appendix. Using the results of 10,000 simulations, we constructed a cost-effectiveness acceptability curve by repeating the Monte Carlo simulation multiple times, building a scatter plot of different realizations, and then determining the proportion of those simulations where a given strategy was ‘preferred’ under a range of willingness to pay (WTP) thresholds. A strategy was called ‘preferred’ if it had the highest probability of having the highest net monetary benefit (NMB) of all strategies under consideration. NMB is defined as a difference between a product of WTP and strategy effectiveness minus the costs associated with that treatment strategy [15].

Uncertainty in parameters leads to the possibility of making the wrong treatment decision, which may lead to increased costs and/or worse health benefits. However, the reduction or elimination of uncertainty through additional research comes at a cost. We used novel methodology to quantify the value of future research to reduce uncertainty in efficacy and costs parameters by estimating expected value of perfect information (EVPI). EVPI measures the difference between the total costs associated with the best possible decision that could be made with additional information compared to the potential costs of the best decision that could be made in the absence of any additional information [15].

Since uncertainty surrounded a sizable number of parameters in our analysis, we also estimated the expected value of partial perfect information (EVPPI), which limits this value-based assessment of future research to a subset of a model’s parameters. Importantly, by valuing uncertainty within each group of parameters, EVPPI can help inform the comparative prioritization of future studies. We then estimated the population value-of-information (VOI) by multiplying per person EVPI by the number of persons expected to encounter treatment decisions during the usable time of information. This approach is particularly important when evaluating common procedures such as APM.

### Model Structure

**Fig 1:**
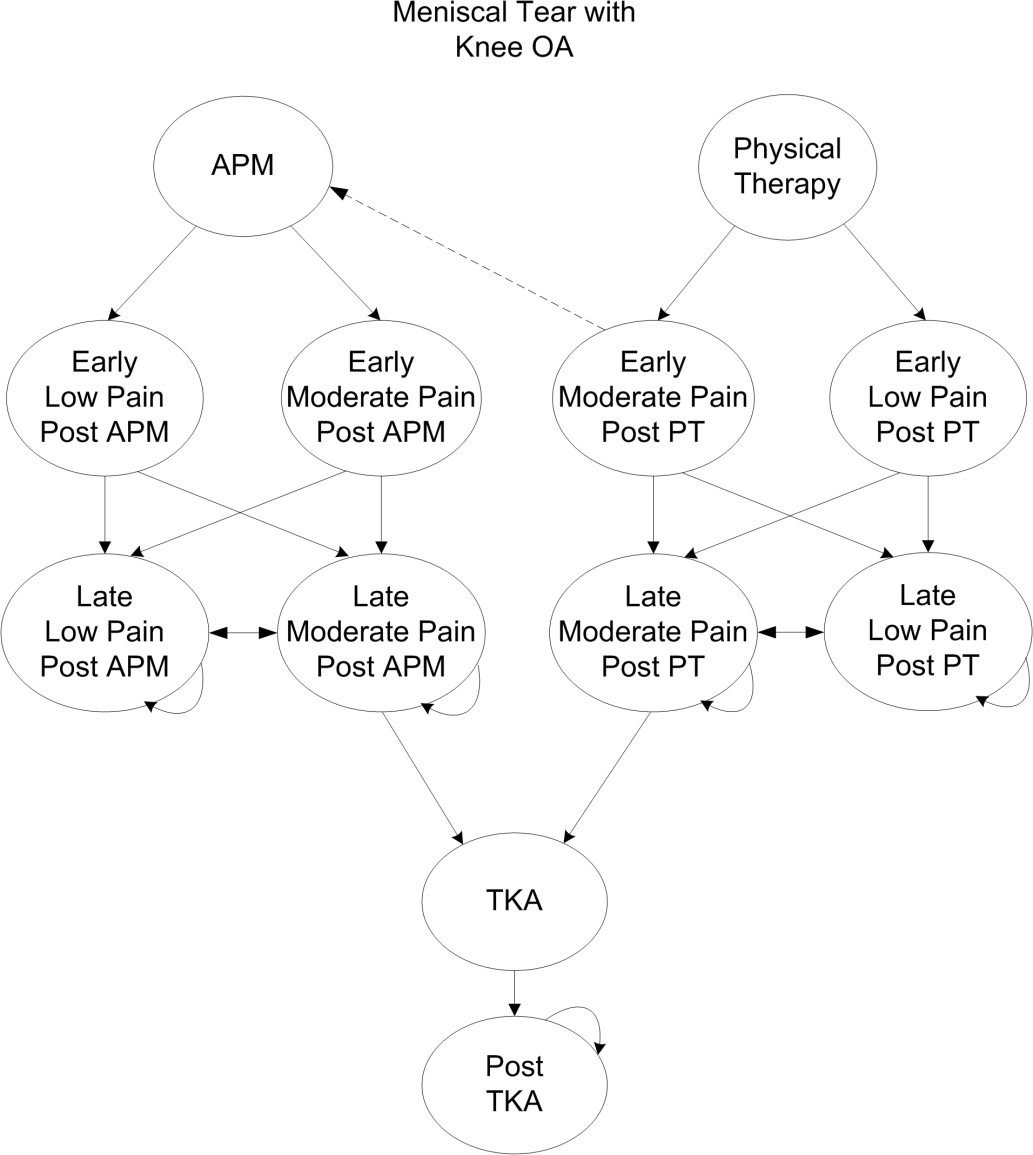
Model Structure for the Treatment of Meniscal Tear. Fig 1 describes the model structure used for evaluating the cost-effectiveness of three strategies used for the treatment of MT in the presence of knee OA: 1) PT, 2) PT with referral for APM in those patients with persistent pain after PT, and 3) APM for all patients. Straight arrows describe a subject’s transition from one health state to another. Curved arrows indicate the possibility of cycling within one health state given no change in pain status. Health states were stratified by KL grade. Subjects who received a particular treatment transitioned through early and late, and low or moderate pain states depending on treatment efficacy and knee OA progression. All subjects in the ‘Delayed APM’ strategy who transitioned to early moderate pain transitioned to APM with probability of 1. Subjects in late pain could transition to TKA.

Subjects moved across the following major health states in the model (Fig 1): 1) initial treatment (APM or PT); 2) early moderate or low pain; and 3) late moderate or low pain. Pain-related health states were stratified by KL grade. Subjects could elect TKA if they had advanced knee OA (KL grades 3 or 4) as well as pain for at least 6 consecutive months after the initial 3-month treatment period. TKA uptake rate was determined according to utilization rates derived from the Multicenter Osteoarthritis Study and Osteoarthritis Initiative cohorts [16]. Death was possible at any state.

### Cohort Characteristics

We generated a cohort reflective of the MeTeOR study population. Subjects entered the simulation model with an average age of 58 and the following distribution of OA severity: 44.8% KL grades 0 or 1, 26.4% KL grade 2, and 28.8% KL grade 3 [6]. Annual mortality was derived from the 2009 intercensal US Life Tables published by the Centers for Disease Control and Prevention [17]. Incidence and structural progression of knee OA were derived from the Johnston County Osteoarthritis Project [18].

### Input Parameters

A substantial number of input parameters were required for the microsimulation. They are presented in Table 1 as well as in the S1 Technical Appendix.

**Table 1:** Base Case Parameters.

**Cohort Characteristics**	** **			**Source**
**Age, Mean (SD)** *	58 (7)			Katz et al. 2013 [6]
**OA Prevalence at Baseline**	** **	**Annual KL Progression**		Katz et al. 2013 [6]; Johnston County Osteoarthritis Project [18]; Holt et al. 2011 [19]
KL grade 0 and 1	44.8%	KL 2 to KL 3	0.0735	
KL grade 2	26.4%	KL 3 to KL 4	0.0267	
KL grade 3	28.8%			
KL grade 4	0.0%			
**Annual OA Incidence, by Age**	** **	**Annual TKA Utilization, by Age**		Losina et al. 2013 [20]; Weinstein et al. 2013 [16]
45–54	0.379%	45–64	0.064	
55–64	0.668%	65–84	0.119	
65–74	0.375%	85+	0.030	
75–84	0.306%	** **		
85+	0.310%	** **		
**Quality of Life Utility Scores**				Katz et al. 2013 [6]
Low Pain (KOOS ≤ 25)	0.869			
High Pain (KOOS > 25)	0.771			
**Pain after MT Treatment** * ‡	**Quarterly Mean by Strategy, PSA Distribution (Alpha, Beta)**			** **
**Months 0 to 3 post Treatment**	**APM**	**APM after PT**	**PT**	Katz et al. 2013 [6]
*Probability of Failed Pain Relief*	* *	* *	* *	
KL grade 0, 1, 2	0.322, Beta (29, 61)	0.400, Beta (6, 9)	0.569, Beta (58, 44)	
KL grade 3, 4	0.488, Beta (21, 22)	0.667, Beta (6, 3)	0.703, Beta (26, 11)	
**Months 3 to 6**				
*Probability of Pain Incidence*				
KL grade 0, 1, 2	0.230, Beta (14, 47)	0	0.227, Beta (10, 34)	
KL grade 3, 4	0.364, Beta (8, 14)	0	0.182, Beta (2, 9)	
*Probability of Pain Resolution*				
KL grade 0, 1, 2	0.483, Beta (14, 15)	0	0.155, Beta (9, 49)	
KL grade 3, 4	0.333, Beta (7, 14)	0	0.115, Beta (3, 23)	
**Knee-OA Related Pain**	**All strategies**		** **	** **
**Annual Pain by KL**	**Pain Incidence**	**Pain Resolution**	** **	Johnston County Osteoarthritis Project [18]
KL grade 0, 1	0.075	0.085		
KL grade 2	0.085	0.037		
KL grade 3	0.212	0.040		
KL grade 4	0.190	0.005		
**TKA, Annual Efficacy**	**Pain Relief**			Katz et al. 2007 [21]
** ** First year	0.862			
** ** Subsequent years	0.960			
**Perioperative Outcomes and Adverse Events (AE)**		** **	** **	** **
**Treatment**	**Probability of AE**	**Mortality, given AE**	** **	
APM	0.015	0.011		Hame et al. 2012 [22]
TKA	0.036	0.006		Katz et al. 2004 [23]
Pharmacologic pain management	0.111	0.005		Goldstein et al. [24]; Silverstein et al. [25]; Solomon et al. [26]
**Costs, Quarterly** ‡	**Mean**	**PSA Distribution**	**Alpha, Lambda**	** **
**Healthcare Costs and Treatment of Pain**				Medicare Fee Schedules [27–29]; Katz et al. 2013 [6]; Red Book Online [30];Healthcare Cost and Utilization Project 2011 [31]; Buntin et al. 2005 [32]
*Cost of APM*	* *	* *	* *	
* * Procedure	$2,867			
* * Complication	$11,589			
* * Healthcare, post-op pain*	$454	Gamma	703, 1.5	
* * PT Rehabilitation*	$439	Gamma	351, 0.8	
*Cost of PT Regimen* *				
Healthcare, post-op pain	$209	Gamma	117, 0.6	
PT Rehabilitation	$568	Gamma	352, 0.6	
*Cost of Pain Management* * *‡‡*	* *	* *	* *	
High Pain cohort	$276	Gamma	129, 0.5	
Low Pain cohort	$99	Gamma	160, 1.6	
Complication	$1,816			
*Cost of TKA†*	* *	* *	* *	
* * Procedure and rehab	$20,282			
* * Complication	$15,149			
**Indirect Costs: Productivity Losses, in hours lost** *				Katz et al. 2013 [6];Bureau of Labor Statistics 2013 [33]
*Months 0 to 3*				
APM	109	Gamma	79, 0.7	
PT	79	Gamma	22, 0.3	
*Months 3 to 6 *				
High Pain	87	Gamma	32, 0.4	
Low Pain	42	Gamma	68, 1.6	
*After 6 months*				
** ** High Pain	70	Gamma	94, 1.4	
Low Pain	30	Gamma	89, 3.0	

* All distributions were sampled in a probabilistic sensitivity analysis (PSA); parameters for these distributions are included in this table.

^†^ Includes total cost of care and rehabilitation following treatment regimen.

‡ All probabilities for changes in pain reported as quarterly probabilities unless otherwise specified

‡‡ Includes cost of select NSAIDs, opioids, acetaminophen, intra-articular injections, visits to the ER, medical appointments, and alternative medicines/therapies.

#### Pain Relief and Incidence

Distributions of pain relief three months after initial treatment (APM or PT) were derived from the MeTeOR study using a transformed Knee injury and Osteoarthritis Outcome Score (KOOS) Pain scale (Table 1). The KOOS scale is a validated measure of knee pain that ranges from 0 to 100, where 100 represents the highest possible level of pain [34,35]. Pain in the model was dichotomized at a KOOS score of 25, with scores above 25 characterized as ‘Moderate Pain’ and scores at or below 25 as ‘Low Pain.’ This threshold was chosen to reflect that scores below 25 correspond to mild pain on a majority of domains on the KOOS scale [35].

Based on the expert opinion of a panel of MeTeOR clinical investigators (RJW, KPS, LAM, MHJ, JNK), we assumed that changes in pain status attributable to a specific treatment should be limited to the first six months following that treatment. Subjects who experienced pain relief at 3 months were able to experience late pain by month 6; similarly, subjects who reported initial failure in pain relief were able to experience pain resolution in the next quarter. Transition probabilities for pain and pain resolution were stratified by OA severity (Table 1). Distributions of the parameters used in our Monte Carlo analysis appear as histograms in S1 Technical Appendix Table A-5.

Six months after initiation of the index treatment, incidence and resolution of knee pain were assumed to be due to changes in subjects’ underlying knee OA. Values associated with the probability of fluctuations in OA-related knee pain were derived from the Johnston County Osteoarthritis Project [18] and were stratified according to KL grade (Table 1).

#### Efficacy of Delayed APM

Due to limited data on the efficacy of APM if pain was not relieved by initial PT, we considered two scenarios for estimating the efficacy of a Delayed APM procedure. In our base case analysis, we used a more conservative scenario and derived a distribution of being in moderate pain after Delayed APM from subjects in the MeTeOR study that were randomized to PT but crossed over to APM three months post-randomization. The 3-month time point for this analysis was chosen in consultation with a number of practicing clinical investigators (both surgeons and physical therapists), who stated that after a three month trial of PT, patients are generally re-evaluated and surgery is considered if PT has not been beneficial. In a sensitivity analysis, we assumed that the efficacy of APM in those who failed PT would be the same as in subjects who had undergone APM initially, as their primary treatment.

#### Quality of Life Estimates

Utilities were derived from the MeTeOR study, where study participants were asked to fill out the EQ-5D instrument [6], a validated measure of QoL across five dimensions of health [36,37]. Health states defined by the EQ-5D descriptive system were matched to a reference set of weights that corresponded to each state’s utility value [38]. These weights were averaged among those with moderate and low pain to create final utility values (Table 1).

#### Adverse Events

Adverse events could occur following APM, TKA, and ingestion of pain medications. Values for the incidence, cost, and mortality associated with these adverse events are shown in Table 1 and the S1 Technical Appendix.

#### Medical Costs

Costs fell into one of two categories: 1) direct medical costs for the treatment of knee pain due to either MT or OA and 2) costs of productivity lost due to treatment and functional disability. These costs appear in Table 1 and are reported in 2013 US Dollars (USD). Utilization of PT sessions for subjects in the PT and the Delayed APM arms were derived from the PT-randomized arm in the MeTeOR trial, while utilization of PT in the Immediate APM arm was derived from the APM-randomized arm in the MeTeOR trial. These costs are the total costs of PT and only applied to the cycles where PT was used.

Subjects also accumulated costs over time due to general medical care, which included scheduled office visits to healthcare providers, trips to the emergency room, and management of knee pain with NSAIDs, opioids, acetaminophen, intra-articular injections, or alternative therapies. Unit costs were derived from Medicare Fee Schedules, Red Book Online, and published literature [27,28,30,39]; a detailed derivation is presented in the S1 Technical Appendix. Utilization of health services and pain control were derived from the MeTeOR trial and stratified by pain severity.

#### Time Costs

Time costs were defined by the number of hours individuals were unable to work as a result of knee pain or time spent undergoing medical care, including surgery, recovery, and PT (Table 1). Time costs also accounted for suboptimal productivity at work. The number of wage-earning hours lost per person in each treatment strategy was taken from the MeTeOR Trial and multiplied by the national mean hourly wage, $22.33/hour [33]. Productivity losses during the first 6 months were derived directly from the data from the MeTeOR trial, and these derivations took into account both employed and unemployed groups. For those who were not employed, we assigned no productivity losses. Overall productivity losses were computed as a weighted average of losses among employed and unemployed. Productivity losses for the longer time frame took into consideration the data from the Bureau of Labor Statistics on the likelihood of employment by age and sex, adjusting subjects’ estimated time costs according to their diminished likelihood of employment over the course of the 10 year time-frame [40]. The derivation of these time costs appears in the S1 Technical Appendix (Tables A-4a and A-4b).

### Analyses

#### Base Case

In the base case analysis, we assumed that APM did not influence OA progression. We did not include time costs. We used conservative estimates of Delayed APM efficacy derived from MeTeOR subjects who crossed over from PT to the APM arm between 3 and 6 months

#### Sensitivity Analysis

We conducted sensitivity analyses that considered alternative scenarios regarding the efficacy of Delayed APM and inclusion of time costs. We conducted a two-way sensitivity analysis in which we simultaneously varied two model parameters identified as critical for the treatment decision-making process by MeTeOR investigators: 1) the potential impact of APM on OA progression and 2) the potential impact of delaying the surgical procedure on its overall efficacy (S1 Technical Appendix). We also conducted a sensitivity analysis reducing the time horizon of analysis from 10-years to 5-years.

### Ethics Approval

This study was approved by the Partners Human Research Committee, the institutional review board of the parent organization of Brigham and Women’s Hospital. All patients in the MeTeOR trial gave written informed consent for their clinical data to be used in this study [41].

## Results

### Base Case Analysis

**Fig 2:**
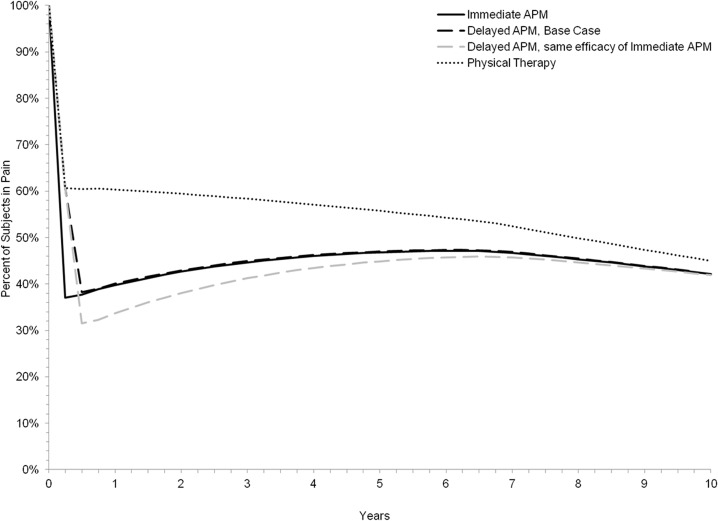
Percentage of Subjects in Moderate Pain, Stratified by Treatment Arm. Fig 2 describes the percentage of subjects reporting pain within each of the evaluated three treatment strategies over the course of 10 years. Two trajectories are reported for the Delayed APM strategy, represented in black and gray dashed lines in the graph. The black dashed Delayed APM trajectory reflects the base case, where the surgery’s efficacy was calculated based on results reported by MeTeOR subjects who crossed over from the non-operative to the operative arm between months 3 and 6. The gray dashed ‘Delayed APM’ line reflects the sensitivity analysis of Delayed APM, where we assumed the efficacy of a delayed surgery following a failed PT regimen would be equal to that of an APM procedure immediately following a MT diagnosis.

Fig 2 illustrates the proportion of persons in each treatment strategy in moderate pain over the course of 10 years. At 6 months, 60% of subjects in the PT-only treatment strategy experienced moderate pain compared to 38% for both the Delayed and Immediate APM strategies. By five years post-treatment the estimated proportion of persons in moderate pain decreased to 56% for the PT strategy and increased to 47% for both the Delayed and Immediate APM strategies. Cumulative incidence of TKA at 10 years in this population ranged from 15% for the PT-only strategy to 14% for both strategies incorporating APM.

The results of all Monte Carlo simulations are presented in a scatter plot in the S1 Technical Appendix (Fig B-2). Table 2 presents a summary of these averaged results and describes the incremental cost-effectiveness of each strategy. Over 10 years, non-operative treatment led to an estimated 6.637 discounted QALYs compared to 6.723 QALYs for the Delayed APM strategy and 6.732 for the Immediate APM strategy. Subjects who underwent Immediate APM incurred the highest direct medical costs at $12,900; PT and Delayed APM were associated with lower direct medical costs of $10,800 and $11,900, respectively. These resulted in estimated ICERs of $12,900/QALY for Delayed APM compared to PT and $103,200/QALY for Immediate APM compared to Delayed APM.

**Table 2:** Cost-effectiveness of Management Strategies for Meniscal Tear, with Sensitivity Analyses.

*Strategy*	*Costs*	*QALYs*	*ICER*
***Base Case***			
* * *PT*	*$10*,*800*	*6*.*637*	* *
* * *Delayed APM*	*$11*,*900*	*6*.*723*	*$12*,*900*
* * *Immediate APM*	*$12*,*900*	*6*.*732*	*$103*,*200*
***Sensitivity Analysis***			
***Base Case*, *5 year Time Horizon***			
*PT*	*$6*,*100*	*3*.*665*	* *
*Delayed APM*	*$7*,*400*	*3*.*727*	*$20*,*900*
*Immediate APM*	*$8*,*400*	*3*.*736*	*$106*,*900*
***Base Case*, *with Time Costs ***			
*Delayed APM*	*$37*,*600*	*6*.*723*	* *
*PT*	*$38*,*200*	*6*.*637*	*Dominated*
*Immediate APM*	*$38*,*300*	*6*.*732*	*$72*,*200*
***Efficacy of Delayed APM equivalent to Immediate APM*, *Time Costs not included***			
*PT*	*$10*,*800*	*6*.*636*	* *
*Delayed APM*	*$11*,*600*	*6*.*744*	*$7*,*400*
*Immediate APM*	*$12*,*900*	*6*.*731*	*Dominated*
***Efficacy of Delayed APM equivalent to Immediate APM*, *with Time Costs***			*** ***
*Delayed APM*	*$36*,*700*	*6*.*746*	* *
*PT*	*$38*,*200*	*6*.*638*	*Dominated*
*Immediate APM*	*$38*,*300*	*6*.*733*	*Dominated*

### Sensitivity Analyses

#### Inclusion of Time Costs

Time costs represented a substantial component of total costs and amounted to $27,400 for the non-operative (PT) strategy, $25,700 for the Delayed APM strategy, and $25,400 for the Immediate APM strategy. With the inclusion of time costs, Delayed APM became cost-saving (i.e. more effective and less expensive) compared to PT alone. Including time costs made Immediate APM more cost-effective than in the base case analysis, with an ICER of $72,200/QALY compared to Delayed APM.

#### Efficacy of Delayed APM

When the efficacy of Delayed APM was made equivalent to that of immediate surgery, Delayed APM produced lower direct medical costs ($11,600) and higher QALYs (6.744) than in the base case analysis, generating an ICER of $7,400/QALY compared to PT (Table 2). Immediate APM became dominated by the Delayed APM strategy, i.e., more expensive with fewer improvements in QALYs. With time costs, Delayed APM became cost-saving compared to PT while Immediate APM remained dominated.

#### Uncertainty around Cost-Effectiveness and Determination of Preferred Treatment Strategies

**Fig 3:**
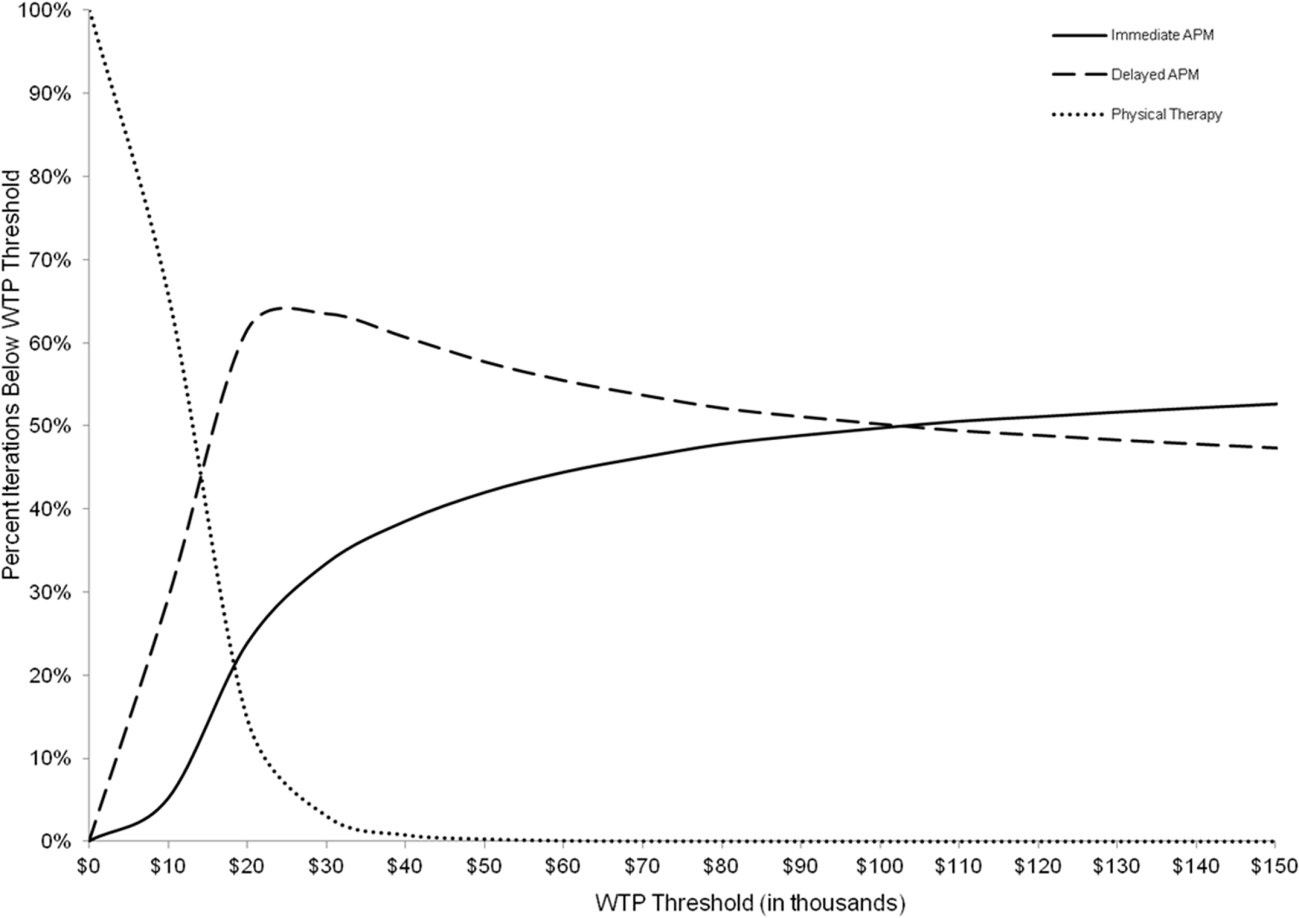
Cost-Effectiveness Acceptability Curve. Fig 3 shows the proportion of iterations where a given strategy proved to be the most cost-effective (i.e., the strategy with the highest NMB whose ICER was below the WTP threshold), represented by the y-axis, given a specific WTP, represented by the x-axis. Time costs were not included.

**Fig 4:**
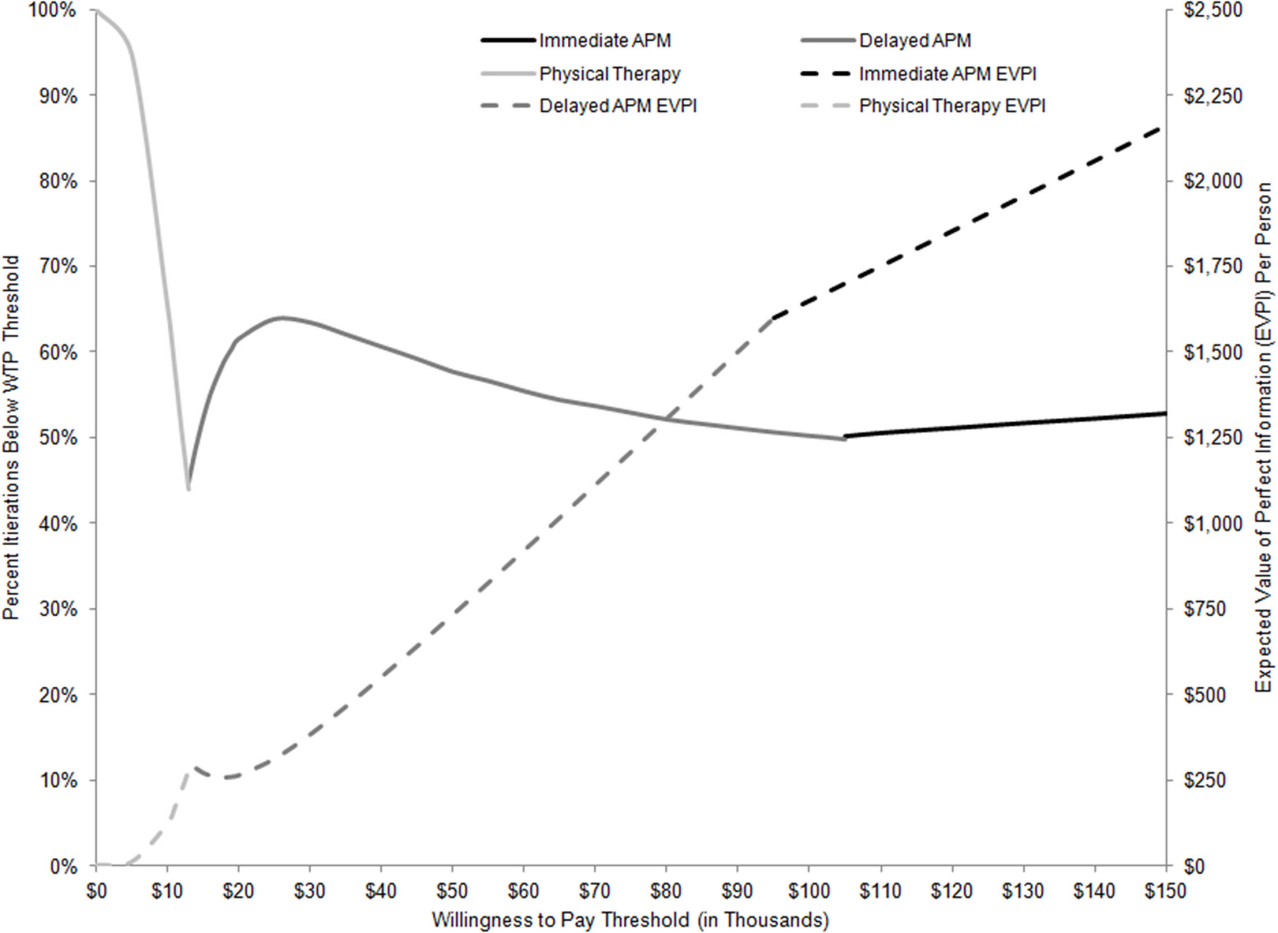
Cost-effectiveness Acceptability Frontier and Expected Value of Perfect Information. Fig 4 contains two categories of reported results. The first is the cost-effectiveness acceptability frontier, described by solid gray and black lines at the top half of the graph. The frontier describes the likelihood that the strategy with highest NMB at any given WTP threshold is cost-effective, where likelihood is defined as a probability on the left-most Y axis. NMB is calculated by subtracting the cost of a treatment strategy from the product of a strategy’s effectiveness and a given WTP. The bottom half of the graph describes the EVPI reported for each WTP threshold for the strategy defined as preferred under that threshold. EVPI results are represented by dotted lines in dollars per person by the right-side Y axis. Time costs were not included.

Fig 3 displays a cost-effectiveness acceptability curve that describes the proportion of iterations for which each strategy was cost-effective at a given WTP threshold. Fig 4 presents a cost-effectiveness acceptability frontier. For the base case scenario with WTP values below $13,000/QALY, PT alone was the preferred strategy because it yielded the greatest NMB the highest number of times. Beyond that WTP threshold, PT was no longer preferred because Delayed APM now had the highest number of iterations with the highest NMB.

At a WTP threshold of $100,000/QALY, Delayed APM was the preferred treatment strategy with 50.2% certainty (i.e. choosing what the model described as ‘preferred’ would ultimately prove not to be cost-effective 49.8% of the time). At a WTP of $103,000/QALY, Immediate APM was preferred. Its probability of being preferred did not exceed 53%, even at a WTP of $150,000/QALY.

Fig 4 also displays EVPI as the quantification of the consequences of making a less-than-optimal (in terms of losses in QALYs and increased costs) treatment choice. EVPI was estimated at $734 per person (pp) at WTP $50,000/QALY ($1,649pp at WTP $100,000/QALY). This implies that by eliminating all uncertainty, we can expect an improvement in net monetary benefit of $734pp ($1,649pp) or that the current expected harm due to uncertainty is $734pp ($1,649pp) with a health equivalent of 5.4 quality-adjusted days (6.0 days). Given that about 352,000 persons between the ages of 45 and 64 years undergo APM every year [2], eliminating all uncertainty could lead to an additional 5,164 QALYs (5,804 QALYs) in this population. Assuming a period of 3 years is the usable life of information resulting from efforts to reduce uncertainty and a discount rate of 3%, we estimate that the maximum benefit from additional research designed to eliminate uncertainty related to benefits and costs of treatment of MT in the presence of OA to be $752.3 million at $50,000/QALY WTP ($1.691 billion at $100,000/QALY WTP).

S1 Technical Appendix Fig B-4 describes the impact of individual parameters on model uncertainty and presents a summary of EVPPI estimates for four key parameters. Of these, the efficacy of a delayed APM generated the highest EVPPI value of $376pp compared to $5pp and $1pp for the impact of APM on OA progression and pain management costs, respectively, given a WTP of $50,000/QALY. With time costs included, EVPPI for time costs was estimated at $59pp.

## Discussion

We examined the cost-effectiveness of three strategies to treat symptomatic MT and OA: PT, PT followed by APM for patients who do not respond to PT, and immediate APM for all patients as a first-line treatment. Despite the fact that several RCTs failed to document the superiority of APM over non-operative treatment or placebo, our results suggest considerable uncertainty surrounding the question of which treatment is actually preferred from a cost-effectiveness standpoint. We found that PT alone was unlikely to be a preferred cost-effective strategy and that the current state of evidence is not sufficient to reject APM on cost-effectiveness grounds in all persons with MT and OA.

Sensitivity analyses demonstrated that as the effectiveness of Delayed APM improved, Immediate APM was associated with greater comparative costs and less comparative effectiveness. Upon quantifying the value of perfect information, we found that the maximum amount society would be willing to pay for better information was $752.3 million. This amount greatly exceeds the cost of conducting another randomized controlled trial; indeed, the cost of conducting the MeTeOR trial was less than $4 million.

Inclusion of time costs generally improved the value of both Delayed APM and Immediate APM since these strategies reduced the time subjects spent in pain, minimizing productivity losses over time. In fact, treatment-related costs represented only a small proportion of the total costs subjects accumulated, as time costs more than *tripled* the total costs incurred over ten years.

To the best of our knowledge, this is the first study to examine the economic implications of treatment strategies for symptomatic MT in the setting of knee OA and the first to quantify uncertainty in treatment decisions. Other studies that have evaluated the cost-effectiveness of knee arthroscopy [42] and anterior cruciate ligament (ACL) reconstruction [42–46] have reported ICERs well below $50,000/QALY for both procedures. While one analysis [45] used Monte Carlo simulation modeling to evaluate the effect of parameter uncertainty on results, the remainder based their findings on deterministic analyses.

The results of this analysis should be interpreted in view of certain limitations. Importantly, our analysis was conducted in mid-to-late 50 year olds with both MT and OA. Accordingly, these results should be generalized cautiously and may not be applicable for subjects reporting MT without OA, for subjects presenting with MT in their 30s or 40s (particularly those who might not be eligible for TKA), or for subjects reporting MT with end-stage (KL 4) OA, which was an exclusion criteria in the MeTeOR trial and hence not modeled in our analysis (patients with end-stage knee OA have poor prognosis for knee arthroscopy). We did not conduct the analysis separately for men and women. Since data from the MeTeOR trial did not reveal significant differences in outcome by gender or age, we chose not to conduct separate subgroup analyses. Should such outcome differences be observed elsewhere, it may prove useful to assess the cost-effectiveness of gender- and age-specific treatment strategies.

Moreover, while key efficacy parameters were derived from one large RCT, additional parameters related to OA pain over the 10 year time span were derived from larger long-term population-based studies. The large uncertainty underlying some key parameter estimates, such whether APM is as efficacious after a failed course of PT as it is soon upon diagnosis or whether APM affects the progression of OA, lends uncertainty to the estimated cost-effectiveness of the Delayed APM strategy. Our multiple sensitivity analyses examine the potential influence varying these important parameters may have on the robustness of our cost-effectiveness results. While we consistently find that Immediate APM is the most expensive strategy, it was only dominated in a sensitivity analysis where the efficacies of Delayed and Immediate APM were set as equal. Lastly, the wage rate is a defensible proxy for the opportunity cost of time only if we assume that the patient population is composed entirely of employed persons. In MeTeOR, about 60% of study participants were employed, with rates similar across both arms. Therefore, we likely underestimated the opportunity costs due to work-related absenteeism.

This work has critical implications for research, clinical care, and policy. While several large RCTs showed no superiority of APM compared to non-surgical or sham procedures, the impact of these trials on clinical practice may be limited. For example, the lack of superiority of APM compared to sham surgery does not illuminate a preferred treatment since sham is not used clinically. While substantial cross-over rates reported by other trials suggest that PT followed by APM if necessary (Delayed APM) may be a key clinical strategy, we know of no head-to-head studies comparing the effectiveness of that strategy and Immediate APM. While only a portion of patients will undergo surgery in the Delayed APM strategy, we kept it as a separate strategy because if APM were to be eliminated as a treatment option for persons with knee OA and MT, the Delayed APM strategy would be eliminated as well. These limitations in the application of trial results to clinical practice emphasize the crucial role of model-based evaluations in aiding medical decision-making.

Our results complement the results of recent RCTs by defining societal WTP thresholds at which surgical strategies may become preferred treatments. Moreover, we have quantified the amount of uncertainty surrounding these choices and offered a framework for prioritization of future research. Model-based evaluations of treatments for persons with MT and OA may help to frame policy recommendations and define research priorities regarding the optimal use of APM-based strategies.

## Supporting Information

S1 Technical Appendix The Technical Appendix provides additional details on the methodology, parameter derivation and reports additional results from sensitivity analyses. (DOCX)
