# Supplementary material for: Defining the Value of Future Research to Identify the Preferred Treatment of Meniscal Tear in the Presence of Knee Osteoarthritis
Source: PLoS One. 2015 Jun 18;10(6):e0130256. doi: 10.1371/journal.pone.0130256 (PMC4472814; doi:10.1371/journal.pone.0130256)
Supplement: S1 Technical Appendix — (DOCX) [file pone.0130256.s001.docx]

**Technical Appendix**

Defining the value of future research to identify the preferred treatment of meniscal tear in the presence of knee osteoarthritis

This Technical Appendix provides additional details on the methodology, parameter derivation and reports additional results from sensitivity analyses.

In Section A, we describe detailed information on the derivation of input parameters, including direct and indirect (time) costs and toxicities associated with the treatment of meniscal tear (MT) in the setting of knee osteoarthritis (OA). All costs in our model were inflated to 2013 US dollars (USD) using data from the Bureau of Labor Statistics on the Annual Consumer Price Index (CPI) [[1](#_ENREF_1)].

In Section B, we describe our further investigation of the impact of uncertainty of our results as well as the impact of including time costs on the comparative cost-effectiveness of our three strategies.

**Section A. Methodology**

All costs herein mentioned were inflated to 2013 US dollars (USD) using the Bureau of Labor Statistics on Annual Consumer Price Index (CPI) [[1](#_ENREF_1)].

**I. Direct Medical Costs**

**A. Complications due to APM**

Complications associated with receiving arthroscopic partial meniscectomy (APM) were identified using results described by Hame *et al*. [[2](#_ENREF_2)] In their investigation of adverse events following APM, the authors evaluated over 300,000 patients who had undergone procedures coded as either CPT-29880 (medial and lateral meniscectomy) or CPT-29881 (medial or lateral meniscectomy) and had experienced an adverse event within 90 days of the treatment. The investigators listed three common complications and their relevant ICD-9-CM codes in their results: pyogenic arthritis (ICD-9 code 711.06), deep venous thrombosis (codes 453.4, 453.41, and 453.12), and pulmonary embolism (415.11). The reported incidence rates for each adverse event were 0.4%, 0.8%, and 0.3%, respectively. We assumed these three complication types represented the majority of complications following APM and used only these event types to derive an overall probability of complication due to APM as well as a value for the cost of these complications. We derived an overall probability of complication by converting these individual incidence rates to probabilities and by summing them; this overall probability of complication was estimated at 0.0150 (Technical Appendix Table A-1).

Mortality associated with APM was derived by aggregating across the probability of death due to each adverse event. We used Healthcare Cost and Utilization Project (HCUP) 2011 data for each ICD-9-CM code to determine the number of discharges among Medicare payers for each code as well as the proportion of Medicare payers who died upon entering the hospital with these events.[[3](#_ENREF_3)] We first calculated a weighted average of mortality for each adverse event based on the relative proportion of discharges reported for a particular code (i.e. the number of discharges associated with 453.4 as compared to 453.14 or 453.12). We then estimated a weighted probability of mortality according to the relative weight of a particular adverse event given an APM complication (e.g. the relative weight of developing deep venous thrombosis among the three possible complications).

Cost of an APM-related complication was similarly derived (Technical Appendix Table A-1). The mean cost per Medicare payer associated with each code was determined according to HCUP 2011 data [[3](#_ENREF_3)].

| **Table A-1. Perioperative Outcomes for APM** | | | | | |  |  |
| --- | --- | --- | --- | --- | --- | --- | --- |
| **Probability of Adverse Event (AE)** | | | | | | | **Sources** |
| **Complication** | **ICD-9 Code** | **Rate of Complication** | | **Probability of AE** | | **Summed probability** | **Mortality** |
| Pyogenic Arthritis | 711.06 | 0.4% | | 0.0040 | | **0.0150** | Hame *et al*. 2012 [[2](#_ENREF_2)]; HCUP 2011 [[3](#_ENREF_3)] |
| Deep Venous Thrombosis | 453.40 | 0.8% | | 0.0080 | |  |  |
|  | 453.41 |  |  |  |  |  |  |
|  | 453.42 |  |  |  |  |  |  |
| Pulmonary Embolism | 415.11 | 0.3% | | 0.0030 | |  |  |
| **Mortality due to Adverse Event** | | | | | | | **Sources** |
| **Complication** | **ICD-9 Code** | **Discharges:**  **Medicare Payers** | **Relative Proportion of Discharges** | **In Hospital Deaths** | **Deaths Weighted by Discharges** | **Mortality Weighted by Relative Probability of AE** | **Mortality** |
| Pyogenic Arthritis | 711.06 | 5767 | 100% | 1.21% | 1.21% | **0.0111** | Hame *et al*. 2012 [[2](#_ENREF_2)]; HCUP 2011 [[3](#_ENREF_3)] |
| Deep Venous Thrombosis | 453.40 | 16,644 | 21% | 1.12% | 0.90% |  |  |
|  | 453.41 | 47,197 | 59% | 0.86% |  |  |  |
|  | 453.42 | 16,085 | 20% | 0.79% |  |  |  |
| Pulmonary Embolism | 415.11 | 2,939 | 100% | 1.53% | 1.53% |  |  |
| **Cost of Adverse Event due to APM** | | | | | | | **Sources** |
| **Complication** | **ICD-9 Code** | **Discharges: Medicare Payers** | **Relative Proportion of Discharges** | **Mean Costs (2013 USD)** | **Costs Weighted by Discharges** | **Mortality Weighted by Relative Probability of AE** | **Cost** |
| Pyogenic Arthritis | 711.06 | 5,767 | 100% | $17,438 | $17,438 | **$11,589** | HCUP 2011 [[3](#_ENREF_3)] |
| Deep Venous Thrombosis | 453.40 | 16,644 | 21% | $7,668 | $8,714 |  |  |
|  | 453.41 | 47,197 | 59% | $9,347 |  |  |  |
|  | 453.42 | 16,085 | 20% | $7,937 |  |  |  |
| Pulmonary Embolism | 415.11 | 2,939 | 100% | $11,440 | $11,440 |  |  |

**B. Cost of Interventions to Treat Meniscal Tear: APM and Physical Therapy**

The direct medical costs associated with the two main interventions used in our four strategies were calculated using a combination of utilization data from the MeTeOR trial in Katz *et al*. 2013 [[4](#_ENREF_4)] as well as Medicare Payment data (Technical Appendix Table A-2) [[5-7](#_ENREF_5)].

The cost of APM was calculated according to Medicare payment data describing different components of the outpatient procedure, including the professional cost components covering physician and anesthesiologist services as well as the technical cost components of the procedure [[5](#_ENREF_5),[7](#_ENREF_7)]. The professional component covering the reimbursement of physician services was derived using the Medicare Physician Fee Schedule 2012 Part B [[5](#_ENREF_5)]. We assumed the vast majority of patients in the MeTeOR cohort underwent surgery in a hospital setting and looked exclusively at mean facility costs within Medicare data. Professional costs associated with reimbursement for the anesthesiologist were derived using the 2013 Medicare Anesthesiology Fee Schedule with HCPCS Code 001400 [[6](#_ENREF_6)]. Time units used for anesthesia were taken from the same database, adjusted in consultation with the senior author, and were calculated into a final cost using the National Anesthesia Conversion Factor from 2013 [[6](#_ENREF_6)]. The cost associated with the technical components of the procedure, including the equipment, operating room, and facility, were derived from the 2012 Medicare Hospital Outpatient Prospective Payment System [[7](#_ENREF_7)].

Costs associated with PT were derived using unit costs for HCPCS codes 97001 and 91110 for Patient Evaluations and Therapeutic Exercises, respectively. We assumed all subjects received a PT evaluation on their first visit and then two units of Therapeutic Exercise per subsequent PT session, as was recommended upon consultation with a physical therapist author from Katz *et al*. 2013 [[4](#_ENREF_4)]. Unit costs were derived from the Medicare Physician Fee Schedule 2012 Part B using the listed HCPCS codes [[5](#_ENREF_5)]. PT costs were incorporated in all four strategies in the analysis as subjects in the surgical arms received post-operative rehabilitation following APM. Accordingly, average utilization of PT units per treatment arm was determined according to the average utilization of PT among subjects in MeTeOR [[4](#_ENREF_4)].

Because each intervention also incurred costs associated with managing pain and healthcare utilization, a cost of care was derived based on the utilization of physician or surgeon office visits, emergency room visits, or pain management via injections or medications according to data from MeTeOR. The costs associated with these methods of managing pain and accessing healthcare resources are described in the Cost of Knee Pain Management section in this appendix. APM-based Treatment strategies in our analysis included a PT rehabilitation regimen; utilization for this post-operative PT regimen was also derived from the mean utilization of PT among subjects in the surgical arm of the MeTeOR study [[4](#_ENREF_4)].

| **Table A-2. Cost of Arthroscopic Partial Meniscectomy and Physical Therapy** | | | | | |
| --- | --- | --- | --- | --- | --- |
| ***APM Surgical Procedure*** | | | | | **Sources** |
| **Description** | **HCPCS Code** | **Component** | **Medicare Payment (Hospital Facility)** | **Total Cost of APM** | **Professional** |
| Arthroscopic medial or lateral meniscectomy | 29881 | Professional - Surgeon | $564 | **$2,867** | Medicare Physician Fee Schedule 2012B [[5](#_ENREF_5)];  Medicare Hospital Outpatient Prospective Payment System 2012[[7](#_ENREF_7)]; Anesthesiology Fee Schedule 2013 [[6](#_ENREF_6)] |
|  |  | Professional - Anesthesia | $175 |  |  |
|  |  | Technical | $2,128 |  |  |
| ***Physical Therapy Unit Costs*** | | | | | **Sources** |
| **Description** | **HCPCS Code** | **Unit cost** | **Units per PT Session** | **Cost of PT Sessions** | **Unit Costs** |
| Initiation PT evaluation | 97001 | $75 | 1 | **$75** | Medicare Physician Fee Schedule 2012B [[5](#_ENREF_5)] |
| Therapeutic exercises | 97110 | $31 | 2 | **$63** |  |
| ***Final Intervention Costs, with Post-Op Care and Rehabilitation*** | | | | | **Sources** |
| **Description** | **Cost of Procedure** | **Cost of PT** | **Cost of Care*** | **Total Treatment Costs** | **Cost of Care** |
| APM | $2,867 | $439 | $454 | **$3,760** | Calculated from derivations in Technical Appendix Table A-3 |
| Physical Therapy | N/A | $568 | $209 | **$777** |  |
| *See costs associated with pain management and healthcare utilization in Technical Appendix Table A-3 | | | | | |

**C. Cost of Knee Pain Management**

Throughout their time in the model, subjects incurred costs associated with managing their ongoing knee pain. These costs of pain management can be divided into two main categories: 1) costs associated with the healthcare resources utilized to receive further care, including appointments with physicians or visits to the ER, or 2) costs associated with specific methods for reducing pain, such as the use of non-steroidal anti-inflammatory drugs (NSAIDs), opioids, injections, or alternative therapies such as acupuncture. For subjects’ first three-month quarter in the model following initial treatment, costs associated with pain management and healthcare resource utilization were calculated according to data described in Katz *et al*. 2013 for subjects in the operative and non-operative arms of the study [[4](#_ENREF_4)]. These utilization data were used to estimate costs of care associated with both APM as well as PT, the estimated values of which appear in Table A-2. For subsequent cycles in the model, subjects incurred a cost associated with moderate pain or low pain according to the mean use of pain management and healthcare resource utilization reported in the combined arms of the MeTeOR study. The values for the quarterly costs associated with reporting moderate pain or low pain appear in the main manuscript in Table 1. The derivations of the costs associated with these methods of pain management and healthcare utilization are show in Technical Appendix Table A-3 and described in detail below.

**i. Office Visits**

Physician office visits included the following three categories: appointments with 1) surgeons, 2) non-surgeon physicians, and 3) physician assistants (PA) and nurse practitioners (NP). Surgeon visits were assumed to occur for subjects who were new or previously established with the surgeon’s office at a ratio of 1 to 2, as estimated by the senior author. Accordingly, the cost of surgeon visits were calculated as a weighted average of the two HCPCS codes 99203 and 99213, which describe office visits for new patients and established patients, respectively. Non-surgeon physician visits as well as PA and NP visits were assumed to all be visits with established patients (HCPCS code 99203).

Costs within each visit type were derived from non-hospital as well as hospital data and assumed subjects saw healthcare providers equally in both settings. Costs associated with non-hospital visits were derived from the Medicare Physician Fee Schedule 2012 [[5](#_ENREF_5)]. The costs associated with visits in hospital settings were estimated by calculating the professional and technical components of the visit cost. This professional component, which included physician reimbursement, was derived using the Medicare Physician Fee Schedule 2012 Part B [[5](#_ENREF_5)] and the technical component of the visit, which included the equipment and facility used, was taken from the October 2012 Hospital Outpatient Prospective Payment System (HOPPS) Addendum B [[7](#_ENREF_7)].

The professional cost component of PA and NP visits was reduced to 85% of the original physician profession cost, as indicated by the diminished cost modifier applied to PA/NP office visits described in Medicare Information for Advanced Practice Registered Nurses, Anesthesiologist Assistants, and Physician Assistants published by the US Department of Health and Human Services in September 2011 [[8](#_ENREF_8)].

**ii. Emergency Room (ER) Visits**

To calculate the cost associated with a visit to the ER, we created a weighted average of the costs associated with ER visits characterized by a range of medical severity. We assumed patients visiting the ER would present with at minimum moderately severe problems and at maximum highly severe problems that threatened life or physiologic function. We used the HCPCS codes 99283 (presented medical problems were of moderate severity), 99284 (presented medical problems were highly severe but with no immediate threat to life or physiologic function), and 99285 (presented medical problems were highly severe with immediate threat to life or physiologic function) to reflect this range; the frequency of these codes was assumed to reflect a 5:3:2 ratio based on assumptions made with a clinician investigator (JNK). We assumed patients would exclusively utilize hospitals when visiting the ER and according derived costs from Medicare payment data for hospital facilities using the listed HCPCS codes. Physician services for these visits were estimated according to the Medicare Physician Fee Schedule 2012 Part B;[[5](#_ENREF_5)] technical components of the visit were derived from the October 2012 Hospital OPPS Addendum B [[7](#_ENREF_7)].

**iii. Injections**

Subjects in the MeTeOR trial were asked whether they had received any of the following types of intra-articular injections in the course of managing their pain: steroid injections, hyaluronic acid injections, or “other” unlisted injection types. Accordingly, we derived cost estimates for each category of injection and assumed that the cost of “other” injections would be roughly equivalent to that of hyaluronic acid injections. To estimate the cost of both steroid and hyaluronic acid injections, we calculated a cost associated with the process of dispensing the injection itself as well as a cost associated with the drug included in the injection. For this first procedural step we derived costs based on Medicare payment data for both hospital and non-hospital facilities using the HCPCS code 20610 for intra-articular injections in a major joint. Non-hospital facility costs were taken from the October 2012 Hospital OPPS Addendum B [[7](#_ENREF_7)]. Professional costs covering physician services conducted in hospital settings were derived from the Medicare Physician Fee Schedule 2012 Part B [[5](#_ENREF_5)]. The technical component of injections dispensed in a hospital setting was calculated by subtracting the cost of lidocaine 10mg (HCPCS code J2001) and methyprednisolone 40mg (HCPCS code J1030) from the original cost of $182.89 listed in the Hospital OPPS report to correct for double-counting of drug costs (OPPS automatically includes drug costs within the technical component of a procedural cost). Drug costs were derived from the Medicare Drug Fee schedule from 2012 and were selected because of their typical use in a hospital setting in intra-articular injections for OA pain [[5](#_ENREF_5)].

To calculate the cost of steroid injections, we combined this procedural cost with the cost of methyprednisolone using HCPCS code J1030. The cost of hyaluronic acid injections was estimated by combining the injection procedural cost with a simple average of the costs associated with injections using hyalgan/supartz (HCPCS J7321), sodium hyaluronate (Euflexxa, J7323), hyaluronan (Orthovisc, J7324), and hylan G-F 20 (Synvisc One, J7323). All listed injections except for Synvisc One involved one injection of a given dose; Synvisc One costs were calculated as a function of 8 injections [[5](#_ENREF_5)].

**iv. Pharmacologic Pain Management**

We estimated costs associated with pharmacologic pain management by deriving the cost of three classes of pain control medications, including acetaminophen, NSAIDs, and opioids. Drug costs were derived using Red Book Online, an online database operated by Truven Health Analytics that reports the average wholesale price (AWP) of pharmaceuticals based on the strength of the drug sold and the number of doses contained in a given package.[[9](#_ENREF_9)] Costs reported in the Red Book Online database are updated daily and were collected for our cost estimations between March and April 2014.

AWPs often overestimate the cost of prescription medications because they include a substantial markup by the wholesaler in the published price.[[10](#_ENREF_10),[11](#_ENREF_11)] We accordingly decided to use these AWPs to determine a value closer to the actual transactional price of a drug: the average sales price (ASP) [[10](#_ENREF_10),[11](#_ENREF_11)]. The ASP is based on transactional sales between a manufacturer and purchaser of a drug after discounts and rebates, bringing the value closer to what the true cost of a drug may be following a process of production, negotiation, reimbursement, and purchase. However, ASPs are not reported with the same transparency of AWP values [[10](#_ENREF_10),[11](#_ENREF_11)] and accordingly must be derived by discounting AWPs published by databases like Red Book. According to recommendations from the Office of the Inspector General at the Department of Health and Human Services, we discounted reported AWPs for prescription medications by 68% for generics and 26% for branded drugs [[11](#_ENREF_11)]. Over-the-counter (OTC) AWPs were not discounted. For both prescription and OTC drugs, we then took a weighted average of these brand-name and generic ASPs. According to data published by the IMS Institute for Health Informatics, patients were prescribed a generic version 93% of the time given the option of a brand and generic form.[[12](#_ENREF_12)] Data on generic use for OTC drugs were taken from a working paper by Bronnenberg *et al*., which reported relative utilization rates of acetaminophen, naproxen, and ibuprofen compared to their brand-name counterparts [[13](#_ENREF_13)].

Utilization of any pain control medication was determined according to the utilization of pharmacologic pain management in the MeTeOR cohort [[4](#_ENREF_4)]. Subjects in the study were asked 1) whether they used any form of pharmacologic pain management, 2) whether this use of pain management included the consumption of acetaminophen, NSAIDs, and/or opioids, and 3) whether their use of pain control medications was daily or occasional. Annual costs associated with daily use of pain control medications were calculated by calculating a particular drug’s daily cost by the number of days in a year. The annual cost of occasional use of pain control was assumed to be 20% of the annual cost associated with daily-use.

Subjects indicating the use of acetaminophen were assumed to take 500mg of the drug three to four times a day, as determined in consultation with the senior author. The daily cost of an NSAID regimen was calculated as a weighted average of commonly used and prescribed NSAIDs for the management of knee OA pain. These drugs, including celecoxib, diclofenac, prescription ibuprofen, meloxicam, nabumetone, and prescription naproxen, were identified using data from the 2009 Medicare Current Beneficiary Survey (MCBS) [[14](#_ENREF_14),[15](#_ENREF_15)]. Subjects in MCBS who indicated they used an “other” form of NSAID on the survey were assumed to use over-the-counter ibuprofen or naproxen. Dosages for each NSAID were based on the results of the MCBS survey data and adjusted under the guidance of the senior author. To determine a single cost attributable to NSAID use, relative utilization rates were derived for each NSAID from the MBCS data to generate a weighted average of the eight NSAID costs.

To derive an estimated annual cost of opioids, we derived costs associated with the following three analgesics, each of which were listed as examples for subjects in the original MeTeOR questionnaire: codeine, oxycodone (e.g. Percocet and Oxycontin), and hydrocodone (Vicodin) [[4](#_ENREF_4)]. Doses and the relative utilization of each of these three analgesics were determined in consultation with the senior author. Consistent with recent FDA regulations cautioning the over-prescription of acetaminophen with opioid medications, we excluded opioid-acetaminophen combination drugs for codeine and oxycodone (hydrocodone is only sold in a combination form) [[16](#_ENREF_16)]. While we excluded combination tablets, we assumed physicians prescribed an independent regimen of acetaminophen with these opioids, as indicated in Technical Appendix Table A-3. We also excluded long-acting extended release opioids as these medications were assumed to be used for intermittent pain control. Moreover, opioids were assumed to be prescribed only in a generic form.

**v. Pharmacologic Pain Management Monitoring**

Pharmacologic toxicity was based on that of non-selective NSAIDs. Major adverse events included gastrointestinal (GI) [[17](#_ENREF_17),[18](#_ENREF_18)] and cardiovascular [[19](#_ENREF_19)] complications; minor complications included non-life threatening reactions such as nausea [[20](#_ENREF_20),[21](#_ENREF_21)]. The probability of a major GI complication was adjusted to account for our assumption that 20% of subjects [[4](#_ENREF_4)] used gastro-protective agents in combination with an NSAID regimen. In the event of toxicity, subjects had a 3.67% chance of cardiovascular complication and 10.7% chance of GI-related complication; all other complications (85.6%) were assumed to be minor. While minor complications had no associated mortality, major adverse events had a 0.48% probability of death given a cardiovascular or GI-related complication. Mortality was derived using HCUP 2011 data on CCS codes 100 (acute myocardial infarction) and 153 (gastrointestinal hemorrhage). Costs associated with major toxicity were derived with HCUP 2011 data using these same codes; minor toxicity costs were derived using published literature [[22](#_ENREF_22)]. Creating a weighted average of these costs resulted in an estimated $1,816 per incident event [[3](#_ENREF_3)].

Subjects also underwent annual laboratory test monitoring to evaluate their physiologic health or use protective agents to counter possible gastrointestinal toxicities while using acetaminophen or NSAIDs daily for three or more consecutive months. Subjects taking acetaminophen were tested for hepatic dysfunction, while those on NSAIDs underwent labs to evaluate their complete blood count as well as the concentration of electrolytes in their bloodstream. The costs associated with these labs were derived from the Medicare Clinical Diagnostic Laboratory Fee Schedule 2012 for HCPCS codes 80076 (hepatic function panel), 85027 (automated complete blood count), and 80051 (electrolyte panel) [[5](#_ENREF_5)]. Because all labs required the withdrawal of blood from a subject, both sets of labs incurred an added cost associated with venipuncture (HCPCS code 36415). These labs and their utilization among subjects were determined according to Grindrod *et al*. for acetaminophen and MCBS 2009 data for NSAIDs [[14](#_ENREF_14),[15](#_ENREF_15),[23](#_ENREF_23)].

We assumed 20% of subjects using NSAIDs would take a proton-pump inhibitor (PPI) as a gastrointestinal protective agent based on data from the MCBS 2009 cohort [[15](#_ENREF_15),[24](#_ENREF_24)] and consultation with JNK. The costs associated with using either omeprazole or lansoprazole were derived from Red Book Online; those taking a PPI were assumed to use omeprazole 75% of the time [[9](#_ENREF_9)].

**vi. Complementary and Alternative Medicines (CAM)**

We estimated costs associated with commonly utilized CAM therapies, including acupuncture, chiropractics, tai chi, yoga, massage, and supplements. Costs associated with each of these products were derived from a 2009 National Health Statistics Report published by the US Department of Health and Human Services [[25](#_ENREF_25)], which described the cost and national utilization of CAM therapies among adults according to data from the 2007 National Health Interview Survey. Utilization of CAM therapies within the simulated cohort was based on the utilization data reported in the MeTeOR trial [[4](#_ENREF_4)].

| **Table A-3. Cost Components for Medical Care Associated with Knee Pain in 2013 USD** | | | | | | |  |
| --- | --- | --- | --- | --- | --- | --- | --- |
| **i. Office Visits** | | | | | | | **Sources** |
| **Visit Type** | **Patient** | **HCPCS Code** | **Hospital Outpatient**  **Cost** | **Non Hospital**  **Cost** | **Simple Average of Components** | **Weighted average of components** | Medicare Physician Fee Schedule 2012B [[5](#_ENREF_5)]; October 2012 Hospital OPPS Addendum B [[7](#_ENREF_7)]; Medicare Information for Advanced Practice Registered Nurses, Anesthesiologist Assistants, and Physician Assistants [[8](#_ENREF_8)] |
| *Surgeon** | New | 99203 | $174 | $108 | $141 | **$113** |  |
|  | Established | 99213 | $125 | $72 | $99 |  |  |
| *Non-Surgeon Physician* | Established | 99213 | $125 | $72 | $99 | **$99** |  |
| *Physician Assistant and Nurse* | Established | 99213 | $117 | $61 | $89 | **$89** |  |
| **ii. Emergency Room Visits** | | | | | | | **Sources** |
| **Severity of Presented Problem** | **Description** | | **HCPCS Code** | **Hospital Outpatient Cost** | **Utilization** | **Weighted Average** | Medicare Physician Fee Schedule 2012B [[5](#_ENREF_5)]; October 2012 Hospital OPPS Addendum B [[7](#_ENREF_7)] |
| *Moderate* | No immediate threat to life or physiologic function | | 99283 | $201 | 50% | **$304** |  |
| *High* | No immediate threat to life or physiologic function | | 99284 | $342 | 30% |  |  |
| *High* | Immediate threat to life or physiologic function | | 99285 | $504 | 20% |  |  |
| **iii. Injections** | | | | | | | **Sources** |
| ***Drug Class*** | **Component** | **HCPCS Code** | **Description** | **Cost** | **Simple Average of Components** | **Total Cost** | Medicare Physician Fee Schedule 2012 Part B [[5](#_ENREF_5)]; Medicare Drug Fee Schedule 2012 [[5](#_ENREF_5)]; October 2012 Hospital OPPS Addendum B [[7](#_ENREF_7)] |
| *Steroid* | Administration | 20610 | Hospital Outpatient | $234 | $153 | **$157** |  |
|  |  |  | Non Hospital | $71 |  |  |  |
|  | Drug | J1030 | Methylprednisolone | $4 | $4 |  |  |
| *Hyaluronic acid* | Administration | 20610 | Hospital Outpatient | $234 | $153 | **$284** |  |
|  |  |  | Non Hospital | $71 |  |  |  |
|  | Drug | J7321 | Hyalgan/supartz | $95 | $131 |  |  |
|  |  | J7323 | Euflexxa | $155 |  |  |  |
|  |  | J7324 | Orthovisc | $174 |  |  |  |
|  |  | J7325 | Synvisc One | $100 |  |  |  |
| *Other* | *Assumed equivalent to hyaluronic acid injection* | | |  |  | **$284** |  |
| **iv. Pharmacologic Pain Management** | | | | | | | **Sources** |
| ***Drug Class*** | **Analgesic** | **Frequency** | **Annual Average Sales Price** | **Utilization within Drug Class** | **Weighted Annual Cost per Drug** | **Weighted Annual Cost per Regimen** | Red Book Online [[9](#_ENREF_9)]; MCBS 2009 [[15](#_ENREF_15)] |
| *Acetaminophen* | Acetaminophen | 500mg 3.5x daily | $71 | 100% | $71 | **$71** |  |
| *NSAIDs* | Celecoxib | 200mg 1.5x daily | $3,047 | 22% | $667 | **$997** |  |
|  | Diclofenac | 50mg 3x daily | $813 | 12% | $95 |  |  |
|  | Ibuprofen (Rx) | 600mg 2x daily | $69 | 9% | $6 |  |  |
|  | Meloxicam | 7.5mg 1x daily | $467 | 30% | $142 |  |  |
|  | Nabumetone | 500mg 2x daily | $283 | 5% | $13 |  |  |
|  | Naproxen (Rx) | 500mg 1.5x daily | $470 | 13% | $62 |  |  |
|  | Ibuprofen (OTC) | 200mg 6x daily | $149 | 4% | $6 |  |  |
|  | Naproxen (OTC) | 220mg 3x daily | $99 | 4% | $4 |  |  |
| *GI Protective Agents*** | Omeprazole | 20 mg 1x daily | $1,366 | 15% | $205 | **$292** |  |
|  | Lansoprazole | 15 mg 1x daily | $1,742 | 5% | $87 |  |  |
| *Opioids†* | Codeine | 30mg 2.5x daily | $124 | 60% | $117 | **$209** |  |
|  | Acetaminophen | 500mg 3.5x daily | $71 |  |  |  |  |
|  | Oxycodone | 5mg 3x daily | $203 | 20% | $55 |  |  |
|  | Acetaminophen | 500mg 3.5x daily | $71 |  |  |  |  |
|  | Hydrocodone w/acetaminophen | 5mg 3x daily | $183 | 20% | $37 |  |  |
| **v. Pain Management Monitoring for Patients taking Medication Daily for 3 or More Months** | | | | | | | **Sources** |
| ***Drug Class*** | **Description of Monitoring Test** | | **HCPCS Code** | **Unit Cost** | **Monitoring Frequency** | **Annual Cost of Tests** | Grindrod et al. 2010 [[23](#_ENREF_23)]; Medicare Clinical Diagnostic Laboratory Fee Schedule 2012 [[5](#_ENREF_5)]; Red Book Online[[9](#_ENREF_9)] |
| *Acetaminophen* | Routine venipuncture | | 36415 | $3 | 1 test annually | **$15** |  |
|  | Hepatic function panel | | 80076 | $12 | 1 test annually |  |  |
| *NSAIDs* | Routine venipuncture | | 36415 | $3 | 1 test annually | **$23** |  |
|  | Complete blood count, automated | | 85027 | $9 | 1 test annually |  |  |
|  | Electrolyte panel | | 80051 | $10 | 1 test annually |  |  |
| **vi. Complementary and Alternative Medicines (CAM)** | | | | | | | **Sources** |
| **CAM Therapy** | |  | **Unit** |  |  | **Out of Pocket Unit Costs** | Nahin *et al*. 2009 [[25](#_ENREF_25)] |
| Acupuncture | |  | per visit |  |  | **$58** |  |
| Chiropractics | |  | per visit |  |  | **$26** |  |
| Tai Chi and Yoga | |  | per visit |  |  | **$5** |  |
| Massage | |  | per visit |  |  | **$56** |  |
| Nonvitamins, nonmineral, natural products | | | per visit |  |  | **$40** |  |
| *Surgeon visits were calculated as a weighted average of new and established patient visits. We assumed a patient would have 3 visits with a surgeon in one year and weighted the average of new patient visits (1 visit) and established visits (2) accordingly. | | | | | | | |
| **GI Protective agents were assumed to be used among 20% of NSAID users. We assumed 75% of those using GI protective agents took omeprazole. This meant that of all NSAID users, 15% of subjects took omeprazole and 5% took lansoprazole while managing pain. | | | | | | | |
| ^†^Combination opioid-acetaminophen tablets were excluded for codeine and oxycodone; physicians were assumed to prescribe independent acetaminophen and opioid regimens. | | | | | | | |

**D. Costs related to TKA**

The cost of TKA surgery was derived from 2012 Medicare Physician Fee Schedules with the exception of the anesthesia component, which was derived from the 2013 Anesthesiology Fee Schedule [[5](#_ENREF_5),[6](#_ENREF_6)]. Costs associated with a patient’s hospital stay and discharge were estimated using 2011 Medicare Payment Data published on Short-Stay Inpatient Diagnosis Related Groups [[26](#_ENREF_26)]. Cost of post-surgery acute care and rehabilitation were included in these cost calculations and were derived from a 2005 Medicare Payment Advisory Commission Report as well as HCUP 2011.[[3](#_ENREF_3)] These combined costs of surgery, anesthesia, and inpatient and follow-up care generated an estimated cost of $20,282 per TKA.

**II. Indirect (Time) Costs**

Indirect costs were calculated in the model based on the number of wage-earning hours a subject was unable to work due to time spent in treatment, at rehabilitation, or with disability due to pain or functional impairment. These time losses were derived first according to the treatment undergone by a subject (APM or PT) and second according to a subject’s pain status (low or moderate pain) based on data from Katz *et al*. [[4](#_ENREF_4)] For the first quarterly cycle in the model, subjects incurred time losses specific to their mode of MT treatment based on the mean productivity lost by subjects in either the surgical or non-surgical arms of the MeTeOR study. In subsequent cycles, subjects incurred time losses according to the mean productivity losses of those subjects in MeTeOR who reported moderate or low levels of pain, regardless of initial treatment strategy. These time losses and their standard errors are reported in Technical Appendix Table A-4a. Time losses were converted to wages lost using the National mean hourly wage of $22.33 per hour, as reported in the May 2013 National Occupational Employment and Wage Estimates Report from the Bureau of Labor Statistics [[27](#_ENREF_27)].

| **Table A-4a. Derivation of Productivity Losses** | | | | | |
| --- | --- | --- | --- | --- | --- |
|  |  | **Wage-Earning Hours Lost Per Person Per Quarter** | | **Worker Wage Losses Per Person per Quarter** | **Sources** |
| **Time Point** | **Description** | **Mean** | **Standard Error** | **Mean** | Katz *et al*. 2013 [[4](#_ENREF_4)]; Bureau of Labor Statistics [[27](#_ENREF_27)] |
| *Months*  *0 to 3* | APM | 109.0 | 12.3 | $2,433 |  |
|  | Physical Therapy | 79.3 | 16.9 | $1,770 |  |
| *Months*  *3 to 6* | Subjects in Moderate Pain | 87.4 | 15.4 | $1,952 |  |
|  | Subjects in Low Pain | 42.2 | 5.1 | $942 |  |
| *After 6 months* | Subjects in Moderate Pain | 69.7 | 7.2 | $1,556 |  |
|  | Subjects in Low Pain | 30.2 | 3.2 | $674 |  |

The calculation of productivity lost and the time costs incurred depended not only on reported pain status but also on the age of the subject. Because the mean age of our population was 58, we assumed that employment would diminish significantly over the 10 year timeframe of the analysis. Accordingly, we derived rates of employment based on age-based civilian labor force participation rates from 2010 reported the by Bureau of Labor Statistics [[24](#_ENREF_24)]. Our derived rates of employed were stratified by subject age and appear below in Technical Appendix Table A-4b.

| **Table A-4b. Diminishing Rate of Employment, by Age** | | |
| --- | --- | --- |
| ***Age*** | **Rate of Labor Force Participation** | **Sources** |
| *55-59* | 1 | Bureau of Labor Statistics [[24](#_ENREF_24)] |
| *60-61* | 0.853 |  |
| *62-64* | 0.679 |  |
| *65-69* | 0.430 |  |
| *70-74* | 0.246 |  |
| *75-79* | 0.149 |  |
| *80+* | 0 |  |

**Distributions used in Monte Carlo simulation modeling**

In Technical Appendix Table A-5 shown on the following pages, we have graphed these distributions as histograms to indicate the degree of uncertainty present when discussing the comparative cost or efficacy of a given strategy. On the y-axis, we indicate the percent of iterations in which a particular value for a parameter (i.e. cost, probability, or time unit) was sampled in the course of a PSA analysis. The x-axis for all figures represents the unit of the parameter. For example, for pain-based parameters, the x-axis is a probability somewhere between and 0 and 1; for costs and productivity losses, the x-axes are defined as cost in 2013 USD and number of wage-earning hours lost, respectively. The purple consistently refers to the immediate APM strategy, whereas blue and green refer to the PT and delayed APM arms, respectively. Moderate pain cohorts are defined by red histograms whereas low pain cohorts are represented in orange. Parameters were stratified by cycle time-points in the model (Months 0-3, 3-6, or 6+), the relevant strategy, the pain status of the cohort, and KL grade (KL 0-2 versus KL 3-4).

**Section B. Additional Results**

**I. Two-way sensitivity analysis: OA Progression and Diminished Surgical Efficacy**

We conducted a two-way sensitivity analysis of the impact of APM on OA progression versus the diminished efficacy of an APM procedure performed on persons who had previously undergone a failed PT regimen. Impact of APM progression ranged from 0.5 to 3.0, with values <1 indicating the percentage by which OA progression was slowed following the receipt of the procedure and values >1 indicating the percentage by which OA progression increased. The probability of pain three months after a delayed APM procedure was varied from the efficacy associated with an immediate surgical procedure (1.00) to a value 40% more likely to result in pain (1.40). In our base case analysis, APM following an initial failed PT regimen was roughly 30% more likely to result in pain than an immediate procedure to treat MT. Incremental cost effectiveness ratios (ICERs) were calculated by comparing the cost and quality-adjusted life expectancy (QALE) of immediate APM with delayed APM, with delayed APM as the reference.

Results of this two-way sensitivity analysis appear in Technical Appendix Fig. B-1. ICERs were categorized by color according to the willingness-to-pay threshold they satisfied. Technical Appendix Fig. 1a reflected the results of this analysis without time costs; in Technical Appendix Fig. 1b, ICERs were calculated with the inclusion of time costs. Immediate APM cost-effectiveness was not strongly affected by changes in KL progression due to surgery and resulted in favorable ICERs when APM slowed or accelerated KL progression to as low as 50% and as much as 275% of our base case values. When the efficacy of a delayed APM equaled that of an immediate procedure, the immediate APM arm became a dominated strategy; when delayed APM was at least 30% more likely than immediate APM to result in pain, the immediate arm produced favorable ICERs under $100,000/QALY.

**Fig. B-1. Two-Way Sensitivity Analysis of the Impact of APM on OA Progression and Diminished APM Efficacy post Initial PT Failure**

**a) No Time Costs, Immediate versus Delayed APM**

| **ICER  Cost/QALY** | | **Increased Pain Failure after Delayed APM** | | | | | | | | |
| --- | --- | --- | --- | --- | --- | --- | --- | --- | --- | --- |
|  |  | 1.00 | 1.05 | 1.10 | 1.15 | 1.20 | 1.25 | 1.30 | 1.35 | 1.40 |
| **Impact of APM on KL Progression** | 0.50 |  |  |  |  |  |  |  |  |  |
|  | 0.75 |  |  |  |  |  |  |  |  |  |
|  | 1.00 |  |  |  |  |  |  |  |  |  |
|  | 1.25 |  |  |  |  |  |  |  |  |  |
|  | 1.50 |  |  |  |  |  |  |  |  |  |
|  | 1.75 |  |  |  |  |  |  |  |  |  |
|  | 2.00 |  |  |  |  |  |  |  |  |  |
|  | 2.25 |  |  |  |  |  |  |  |  |  |
|  | 2.50 |  |  |  |  |  |  |  |  |  |
|  | 2.75 |  |  |  |  |  |  |  |  |  |
|  | 3.00 |  |  |  |  |  |  |  |  |  |

**b) Time Costs Included, Immediate versus Delayed APM**

| **ICER  Cost/QALY** | | **Increased Pain Failure after Delayed APM** | | | | | | | | |
| --- | --- | --- | --- | --- | --- | --- | --- | --- | --- | --- |
|  |  | 1.00 | 1.05 | 1.10 | 1.15 | 1.20 | 1.25 | 1.30 | 1.35 | 1.40 |
| **Impact of APM on KL Progression** | 0.50 |  |  |  |  |  |  |  |  |  |
|  | 0.75 |  |  |  |  |  |  |  |  |  |
|  | 1.00 |  |  |  |  |  |  |  |  |  |
|  | 1.25 |  |  |  |  |  |  |  |  |  |
|  | 1.50 |  |  |  |  |  |  |  |  |  |
|  | 1.75 |  |  |  |  |  |  |  |  |  |
|  | 2.00 |  |  |  |  |  |  |  |  |  |
|  | 2.25 |  |  |  |  |  |  |  |  |  |
|  | 2.50 |  |  |  |  |  |  |  |  |  |
|  | 2.75 |  |  |  |  |  |  |  |  |  |
|  | 3.00 |  |  |  |  |  |  |  |  |  |

| -5 | Dominated |
| --- | --- |
|  | ICER > $144,000/QALY |
|  | ICER = $100,000 to $144,000/QALY |
|  | ICER = $50,000 to $100,000/QALY |
|  | ICER = $25,000 to $50,000/QALY |
|  | ICER < $25,000/QALY |

**II. Additional Results of Monte Carlo Simulations**

As we described in the main manuscript, we conducted a Monte Carlo analysis using 10,000 simulations to examine the impact of uncertainty in our parameters on our outcomes. Using distributions illustrated in Table A-5, we simultaneously drew from the distributions of the following parameters: 1) early (before 6 months) and late (after 6 months) efficacy of the three strategies; 2) direct medical costs related to treatment as well as pain management; and 3) indirect time costs related to lost economic productivity. In Technical Appendix Fig. A-2, we report the outcomes associated with each of these simulations for each strategy in the form of scatter-plots, with cost in 2013 USD on the x axis and quality-adjusted life expectancy (QALE) on the y axis. Technical Appendix Fig. B-2a reflects the results of our analysis with no time costs included; Technical Appendix Fig. B-2b summarizes the results when time costs were included. Larger symbols colored in purple, green, and blue represent the base case (BC) expected values reported in Table 2 of the main manuscript for the immediate APM, delayed APM, and PT strategies, respectively.

Of note, Technical Appendix Fig. B-2b reports a much larger spread of results compared to the clustered clouds of results portrayed in Technical Appendix Fig. B-2a. We suspect this arose both from fact that time costs became such a significant part of total costs accrued as well as from the fact that employment decreased with age in our model. Because some subjects worked very little in the model, they had very few wage-earning hours to lose; accordingly, costs associated with these subjects looked more akin to those accrued when time costs were not included in the analysis (i.e., those in Technical Appendix Fig. B-2a).

**Fig. B-2. Monte Carlo Simulations: Cost Effectiveness Scatter Plot of Expected Values**

**a) No Time Costs**

**
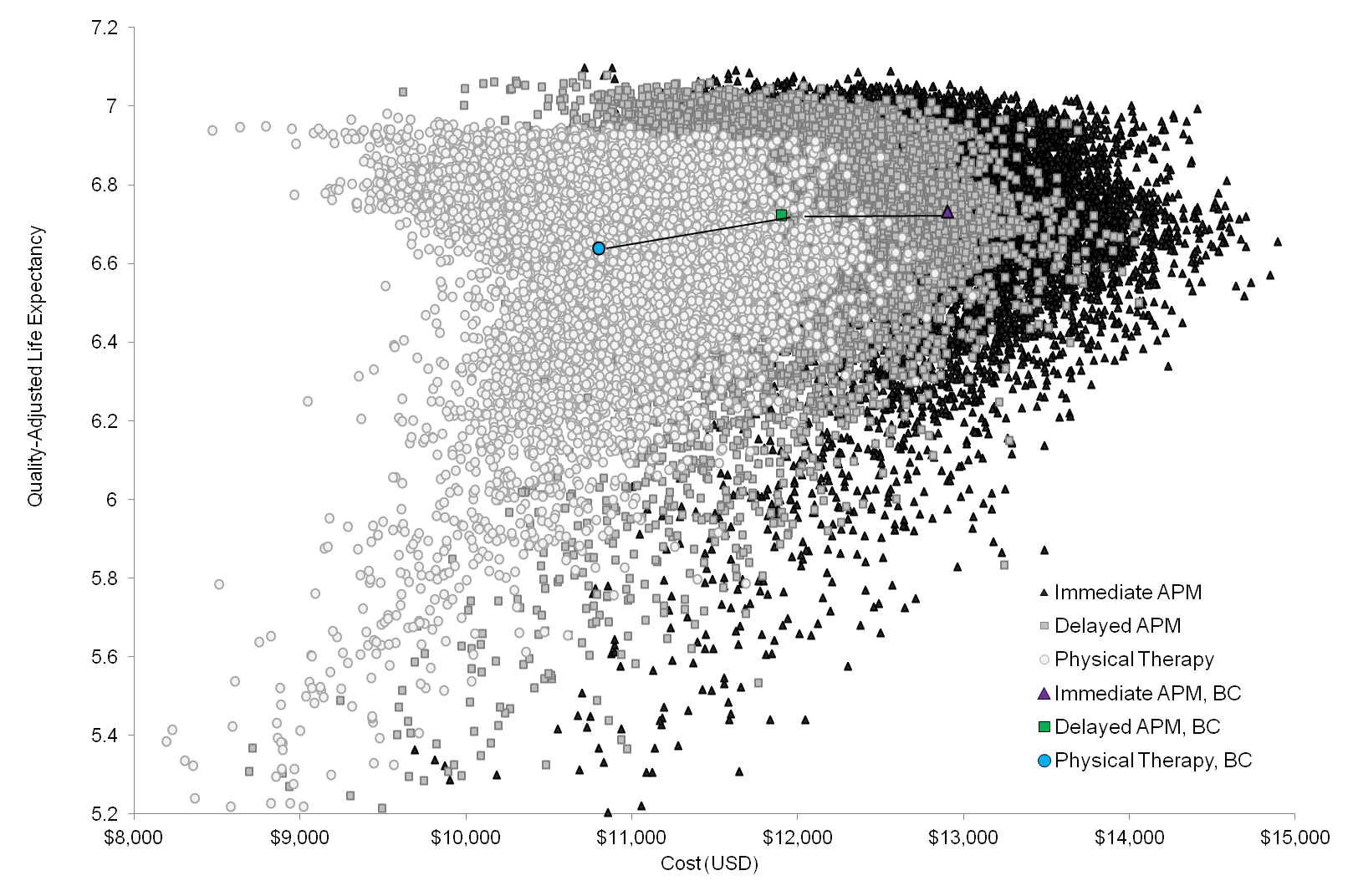
**

**b) With Time Costs**

**
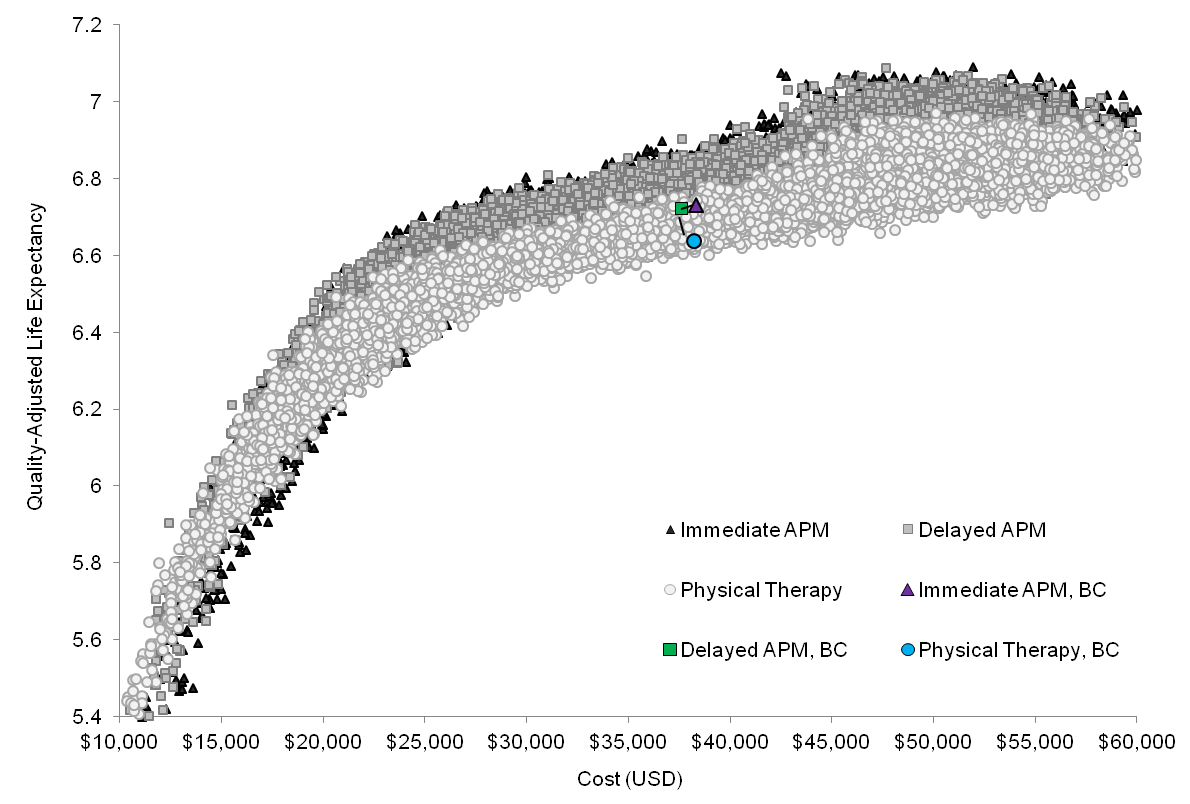
**

The two figures in Technical Appendix Fig. B-3 summarize the impact of uncertainty across our simulations. Technical Appendix Fig. B-3a depicts a Cost-Effectiveness Acceptability Curve for a simulation set with time costs included to demonstrate the percentage of simulations that were cost-effective at a given willingness to pay (WTP) threshold. For example, at a WTP of greater than $20,000/QALY, the likelihood that PT was cost-effective was less than 5%. At a WTP of $90,000/QALY, immediate APM was cost-effective for 51% of our simulations. Consistent with the base case results without time costs, the curves demonstrated a flatness that reflected the influence of uncertainty on our results.

**Fig. B-3a. Cost-Effectiveness Acceptability Frontier: Uncertainty and Value-of-Information Analysis, Time Costs Included**

**
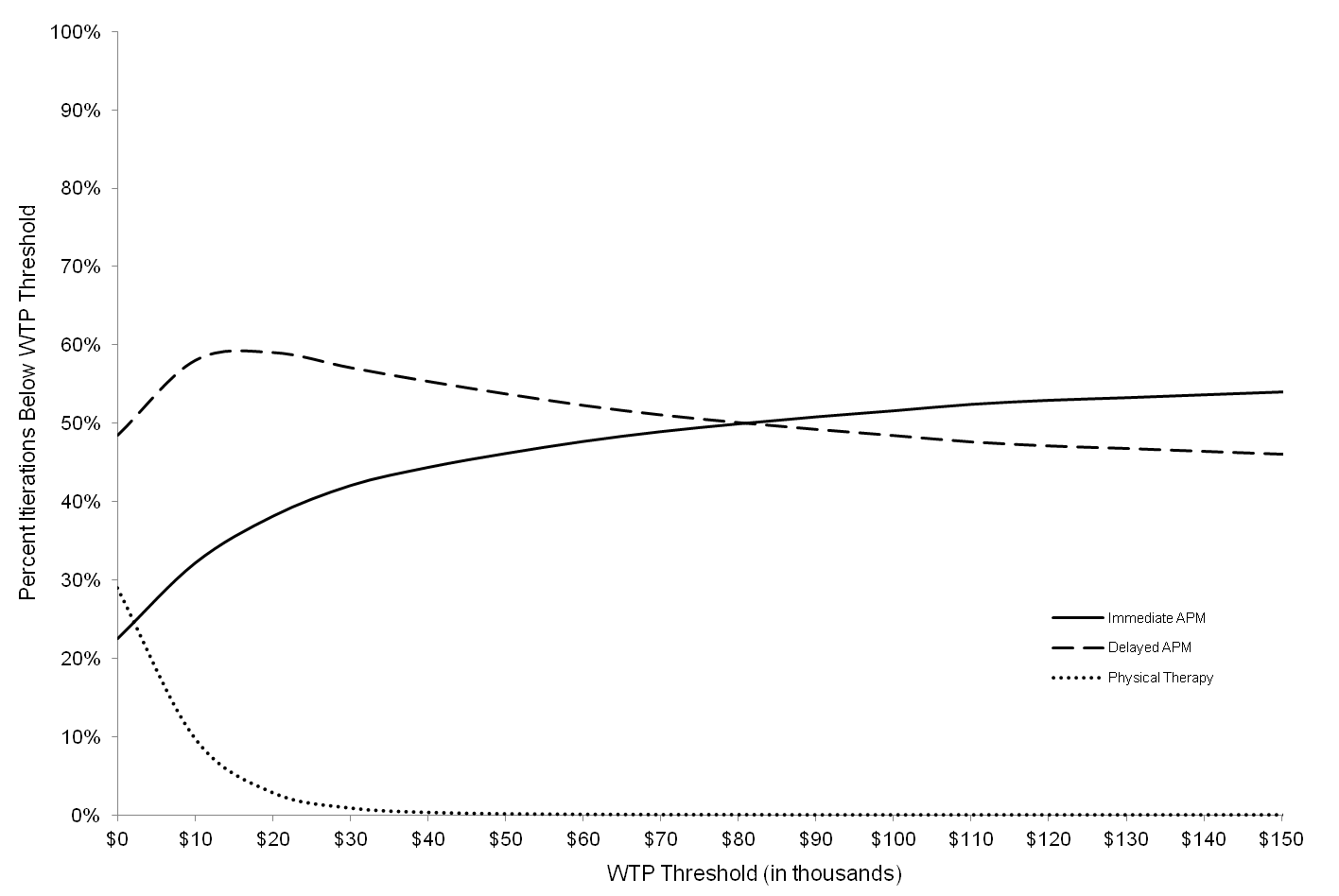
**

Technical Appendix Fig. B-3b shows a Cost-Effectiveness Acceptability Frontier using probabilistic simulations with time costs included. The top half of the figure with solid gray and black lines represents the optimal strategy based on the calculation of NMB. PT was never the preferred strategy at any WTP threshold; delayed APM was most likely to be optimal (59% of the time) at a WTP threshold of $14,000/QALY; at a WTP of $81,000/QALY, immediate APM was preferred with 50% certainty. The bottom half of the graph represents the value of information analysis, where the expected value of perfect information is measured on the right-most Y axis. At a WTP of $50,000/QALY, the value of reducing all parameter uncertainty was lowest within the delayed APM arm at $1,189 per person (pp). For the immediate APM and PT strategies at this threshold, EVPI was estimated at $1,438pp and $6,067pp, respectively.

**Fig. B-3b. Cost-Effectiveness Acceptability Frontier: Uncertainty and Value-of-Information Analysis, Time Costs Included**

**
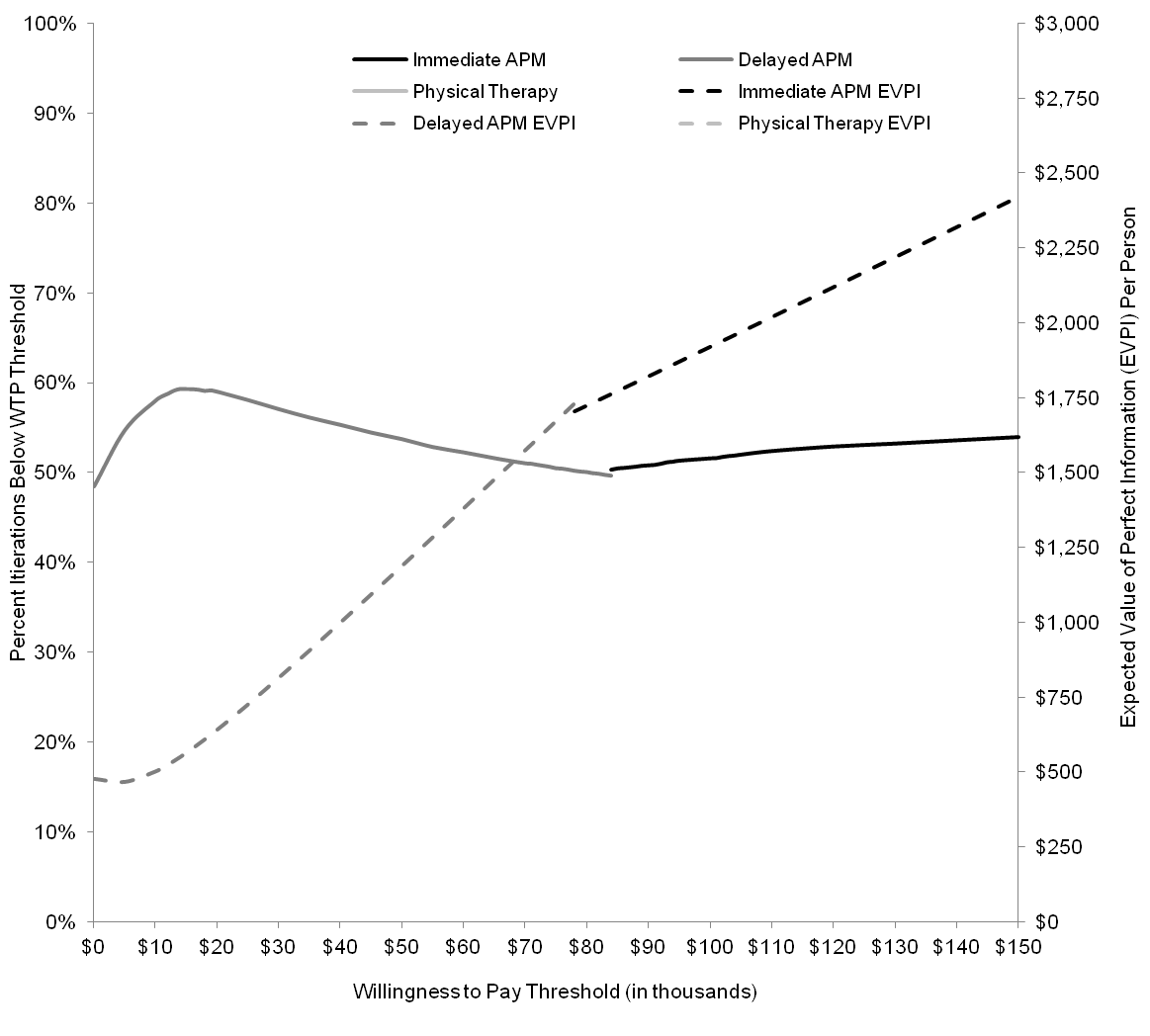
**

Technical Appendix Fig. B-4 summarizes the results of our value of information analysis estimating EVPPI, or the value of reducing uncertainty in a specific subset of parameters. In this figure, the gray columns represent the EVPPI analysis when time costs were included, whereas the black columns reflect the results of an analysis with no time costs. We evaluated the following key parameters: 1) the impact of APM on OA progression, 2) the efficacy of a delayed (compared to immediate) APM procedure, 3) the cost of pain management, and 4) the costs associated with productivity losses. We then estimated the EVPPI associated with these parameters at WTP thresholds of $50,000/QALY and $100,000/QALY. Of the four parameters, early efficacy of delayed APM appeared to be the parameter whose reduction in uncertainty produced the greatest payoff at both WTP thresholds. Because time costs were so substantial, reducing uncertainty in any parameter had the potential to contribute an even greater payoff.

**Fig. B-4. Expected Value of Partial Perfect Information (EVPPI) of Key Parameters**

**References**

1. (2014) Consumer Price Index, 1935-2014. US Bureau of Labor Statistics.

2. Hame SL, Nguyen V, Ellerman J, Ngo SS, Wang JC, Gamradt SC (2012) Complications of arthroscopic meniscectomy in the older population. Am J Sports Med 40: 1402-1405.

3. (2011) Healthcare Cost and Utilization Project. Nationwide Inpatient Sample. Rockville, MD: Agency for Healthcare Research and Quality.

4. Katz JN, Brophy RH, Chaisson CE, de Chaves L, Cole BJ, Dahm DL, et al. (2013) Surgery versus physical therapy for a meniscal tear and osteoarthritis. N Engl J Med 368: 1675-1684.

5. (2012) Medicare Fee Schedules. Center for Medicare and Medicaid Services.

6. (2013) Anesthesiology Fee Schedule 2013. Centers for Medicare & Medicaid Services.

7. (2012) Medicare Hospital Outpatient Prospective Payment System, Addendum B. Centers for Medicare & Medicaid Services.

8. (2011) Medicare information for advanced practice registered nurses, anesthesiologist assistants, and physician assistants. Centers for Medicare & Medicaid Services, Department of Health and Human Services.

9. (2014) Red Book Online. Truven Health Analytics Inc.

10. Curtiss FR, Lettrich P, Fairman KA (2010) What is the price benchmark to replace average wholesale price (AWP)? J Manag Care Pharm 16: 492-501.

11. Levinson DR (2005) Medicaid drug price comparion: average sales price to average wholesale price. Office of Inspector General, Department of Health and Human Services.

12. (2011) The Use of Medicines in the United States: Review of 2010. Parsippany, NJ: IMS Institute for Healthcare Informatics.

13. Bronnenberg BJ, Dubé J-P, Gentzkow M, Shapiro JM (2013) Working paper: Do pharmacists buy Bayer? Sophisticated shoppers and the brand premium. University of Chicago Booth School of Business.

14. Wright EA, Katz JN, Abrams S, Solomon DH, Losina E (2014) Trends in prescription of opioids from 2003-2009 in persons with knee osteoarthritis. Arthritis Care Res (Hoboken) 66: 1489-1495.

15. (2009) Medicare Current Beneficiary Survey. Centers for Medicare & Medicaid Services.

16. (2014) Acetaminophen prescription combination drug products with more than 325 mg: FDA statement. Recommendation to discontinue prescribing and dispensing, 1/14/2014. US Food and Drug Administration.

17. Goldstein JL, Silverstein FE, Agrawal NM, Hubbard RC, Kaiser J, Maurath CJ, et al. (2000) Reduced risk of upper gastrointestinal ulcer complications with celecoxib, a novel COX-2 inhibitor. Am J Gastroenterol 95: 1681-1690.

18. Silverstein FE, Graham DY, Senior JR, Davies HW, Struthers BJ, Bittman RM, et al. (1995) Misoprostol reduces serious gastrointestinal complications in patients with rheumatoid arthritis receiving nonsteroidal anti-inflammatory drugs. A randomized, double-blind, placebo-controlled trial. Ann Intern Med 123: 241-249.

19. Solomon SD, McMurray JJ, Pfeffer MA, Wittes J, Fowler R, Finn P, et al. (2005) Cardiovascular risk associated with celecoxib in a clinical trial for colorectal adenoma prevention. N Engl J Med 352: 1071-1080.

20. Bensen WG, Fiechtner JJ, McMillen JI, Zhao WW, Yu SS, Woods EM, et al. (1999) Treatment of osteoarthritis with celecoxib, a cyclooxygenase-2 inhibitor: a randomized controlled trial. Mayo Clin Proc 74: 1095-1105.

21. Scott DL, Berry H, Capell H, Coppock J, Daymond T, Doyle DV, et al. (2000) The long-term effects of non-steroidal anti-inflammatory drugs in osteoarthritis of the knee: a randomized placebo-controlled trial. Rheumatology (Oxford) 39: 1095-1101.

22. Kamath CC, Kremers HM, Vanness DJ, O'Fallon WM, Cabanela RL, Gabriel SE (2003) The cost-effectiveness of acetaminophen, NSAIDs, and selective COX-2 inhibitors in the treatment of symptomatic knee osteoarthritis. Value Health 6: 144-157.

23. Grindrod KA, Marra CA, Colley L, Cibere J, Tsuyuki RT, Esdaile JM, et al. (2010) After patients are diagnosed with knee osteoarthritis, what do they do? Arthritis Care Res (Hoboken) 62: 510-515.

24. Toossi M (2012) Labor force projections to 2020: a more slowly growing workforce. US Bureau of Labor Statistics. 43-64 p.

25. Nahin R, Barnes P, Stussman B, Bloom B (2009) Costs of Complementary and Alternative Medicine (CAM) and frequency of visits to CAM practitioners: United States, 2007. Hyattsville, MD: National Center for Health Statistics.

26. (2011) Medicare Hospital Inpatient Prospective Payment System. Centers for Medicare & Medicaid Services.

27. (2013) May 2013 National Occupational Employment and Wage Estimates, United States. Washington, D.C.: US Bureau of Labor Statistics, Division of Occupational Employment Statistics.
